# Native diversity buffers against severity of non-native tree invasions

**DOI:** 10.1038/s41586-023-06440-7

**Published:** 2023-08-23

**Authors:** Camille S. Delavaux, Thomas W. Crowther, Constantin M. Zohner, Niamh M. Robmann, Thomas Lauber, Johan van den Hoogen, Sara Kuebbing, Jingjing Liang, Sergio de-Miguel, Gert-Jan Nabuurs, Peter B. Reich, Meinrad Abegg, Yves C. Adou Yao, Giorgio Alberti, Angelica M. Almeyda Zambrano, Braulio Vilchez Alvarado, Esteban Alvarez-Dávila, Patricia Alvarez-Loayza, Luciana F. Alves, Christian Ammer, Clara Antón-Fernández, Alejandro Araujo-Murakami, Luzmila Arroyo, Valerio Avitabile, Gerardo A. Aymard, Timothy R. Baker, Radomir Bałazy, Olaf Banki, Jorcely G. Barroso, Meredith L. Bastian, Jean-Francois Bastin, Luca Birigazzi, Philippe Birnbaum, Robert Bitariho, Pascal Boeckx, Frans Bongers, Olivier Bouriaud, Pedro H. S. Brancalion, Susanne Brandl, Roel Brienen, Eben N. Broadbent, Helge Bruelheide, Filippo Bussotti, Roberto Cazzolla Gatti, Ricardo G. César, Goran Cesljar, Robin Chazdon, Han Y. H. Chen, Chelsea Chisholm, Hyunkook Cho, Emil Cienciala, Connie Clark, David Clark, Gabriel D. Colletta, David A. Coomes, Fernando Cornejo Valverde, José J. Corral-Rivas, Philip M. Crim, Jonathan R. Cumming, Selvadurai Dayanandan, André L. de Gasper, Mathieu Decuyper, Géraldine Derroire, Ben DeVries, Ilija Djordjevic, Jiri Dolezal, Aurélie Dourdain, Nestor Laurier Engone Obiang, Brian J. Enquist, Teresa J. Eyre, Adandé Belarmain Fandohan, Tom M. Fayle, Ted R. Feldpausch, Leandro V. Ferreira, Markus Fischer, Christine Fletcher, Lorenzo Frizzera, Javier G. P. Gamarra, Damiano Gianelle, Henry B. Glick, David J. Harris, Andrew Hector, Andreas Hemp, Geerten Hengeveld, Bruno Hérault, John L. Herbohn, Martin Herold, Annika Hillers, Eurídice N. Honorio Coronado, Cang Hui, Thomas T. Ibanez, Iêda Amaral, Nobuo Imai, Andrzej M. Jagodziński, Bogdan Jaroszewicz, Vivian Kvist Johannsen, Carlos A. Joly, Tommaso Jucker, Ilbin Jung, Viktor Karminov, Kuswata Kartawinata, Elizabeth Kearsley, David Kenfack, Deborah K. Kennard, Sebastian Kepfer-Rojas, Gunnar Keppel, Mohammed Latif Khan, Timothy J. Killeen, Hyun Seok Kim, Kanehiro Kitayama, Michael Köhl, Henn Korjus, Florian Kraxner, Diana Laarmann, Mait Lang, Simon L. Lewis, Huicui Lu, Natalia V. Lukina, Brian S. Maitner, Yadvinder Malhi, Eric Marcon, Beatriz Schwantes Marimon, Ben Hur Marimon-Junior, Andrew R. Marshall, Emanuel H. Martin, Olga Martynenko, Jorge A. Meave, Omar Melo-Cruz, Casimiro Mendoza, Cory Merow, Abel Monteagudo Mendoza, Vanessa S. Moreno, Sharif A. Mukul, Philip Mundhenk, María Guadalupe Nava-Miranda, David Neill, Victor J. Neldner, Radovan V. Nevenic, Michael R. Ngugi, Pascal A. Niklaus, Jacek Oleksyn, Petr Ontikov, Edgar Ortiz-Malavasi, Yude Pan, Alain Paquette, Alexander Parada-Gutierrez, Elena I. Parfenova, Minjee Park, Marc Parren, Narayanaswamy Parthasarathy, Pablo L. Peri, Sebastian Pfautsch, Oliver L. Phillips, Nicolas Picard, Maria Teresa T. F. Piedade, Daniel Piotto, Nigel C. A. Pitman, Irina Polo, Lourens Poorter, Axel D. Poulsen, Hans Pretzsch, Freddy Ramirez Arevalo, Zorayda Restrepo-Correa, Mirco Rodeghiero, Samir G. Rolim, Anand Roopsind, Francesco Rovero, Ervan Rutishauser, Purabi Saikia, Christian Salas-Eljatib, Philippe Saner, Peter Schall, Dmitry Schepaschenko, Michael Scherer-Lorenzen, Bernhard Schmid, Jochen Schöngart, Eric B. Searle, Vladimír Seben, Josep M. Serra-Diaz, Douglas Sheil, Anatoly Z. Shvidenko, Javier E. Silva-Espejo, Marcos Silveira, James Singh, Plinio Sist, Ferry Slik, Bonaventure Sonké, Alexandre F. Souza, Stanislaw Miscicki, Krzysztof J. Stereńczak, Jens-Christian Svenning, Miroslav Svoboda, Ben Swanepoel, Natalia Targhetta, Nadja Tchebakova, Hans ter Steege, Raquel Thomas, Elena Tikhonova, Peter M. Umunay, Vladimir A. Usoltsev, Renato Valencia, Fernando Valladares, Fons van der Plas, Tran Van Do, Michael E. van Nuland, Rodolfo M. Vasquez, Hans Verbeeck, Helder Viana, Alexander C. Vibrans, Simone Vieira, Klaus von Gadow, Hua-Feng Wang, James V. Watson, Gijsbert D. A. Werner, Susan K. Wiser, Florian Wittmann, Hannsjoerg Woell, Verginia Wortel, Roderik Zagt, Tomasz Zawiła-Niedźwiecki, Chunyu Zhang, Xiuhai Zhao, Mo Zhou, Zhi-Xin Zhu, Irie C. Zo-Bi, Daniel S. Maynard

**Affiliations:** 1 Institute of Integrative Biology, ETH Zurich (Swiss Federal Institute of Technology), Zurich, Switzerland; 2 The Forest School at The Yale School of the Environment, Yale University, New Haven, CT USA; 3 Department of Forestry and Natural Resources, Purdue University, West Lafayette, IN USA; 4 Department of Crop and Forest Sciences, University of Lleida, Lleida, Spain; 5 Joint Research Unit CTFC–AGROTECNIO–CERCA, Solsona, Spain; 6 Wageningen University and Research, Wageningen, The Netherlands; 7 Department of Forest Resources, University of Minnesota, St Paul, MN USA; 8 Hawkesbury Institute for the Environment, Western Sydney University, Penrith, New South Wales Australia; 9 Institute for Global Change Biology, and School for Environment and Sustainability, University of Michigan, Ann Arbor, MI USA; 10 Swiss Federal Institute for Forest, Snow and Landscape Research WSL, Birmensdorf, Switzerland; 11 UFR Biosciences, University Félix Houphouët-Boigny, Abidjan, Côte d’Ivoire; 12 Department of Agricultural, Food, Environmental and Animal Sciences, University of Udine, Udine, Italy; 13 Faculty of Science and Technology, Free University of Bolzano, Bolzano, Italy; 14 Spatial Ecology and Conservation Laboratory, Department of Tourism, Recreation and Sport Management, University of Florida, Gainesville, FL USA; 15 Forestry School, Tecnológico de Costa Rica TEC, Cartago, Costa Rica; 16 Fundacion ConVida, Universidad Nacional Abierta y a Distancia, UNAD, Medellin, Colombia; 17 Field Museum of Natural Histiory, Chicago, IL USA; 18 Center for Tropical Research, Institute of the Environment and Sustainability, UCLA, Los Angeles, CA USA; 19 Silviculture and Forest Ecology of the Temperate Zones, University of Göttingen, Göttingen, Germany; 20 Division of Forest and Forest Resources, Norwegian Institute of Bioeconomy Research (NIBIO), Ås, Norway; 21 Museo de Historia Natural Noel kempff Mercado, Santa Cruz, Bolivia; 22 European Commission, Joint Research Center, Ispra, Italy; 23 UNELLEZ-Guanare, Programa de Ciencias del Agro y el Mar, Herbario Universitario (PORT), Portuguesa, Venezuela; 24 Compensation International S. A. Ci Progress–GreenLife, Bogotá, Colombia; 25 School of Geography, University of Leeds, Leeds, UK; 26 Department of Geomatics, Forest Research Institute, Raszyn, Poland; 27 Naturalis Biodiversity Center, Leiden, The Netherlands; 28 Centro Multidisciplinar, Universidade Federal do Acre, Rio Branco, Brazil; 29 Proceedings of the National Academy of Sciences, Washington, DC USA; 30 Department of Evolutionary Anthropology, Duke University, Durham, NC USA; 31 TERRA Teach and Research Centre, Gembloux Agro Bio-Tech, University of Liege, Liege, Belgium; 32 United Nation Framework Convention on Climate Change, Bonn, Germany; 33 Institut Agronomique néo-Calédonien (IAC), Nouméa, New Caledonia; 34 AMAP, University of Montpellier, Montpellier, France; 35 CIRAD, CNRS, INRAE, IRD, Montpellier, France; 36 Institute of Tropical Forest Conservation, Mbarara University of Sciences and Technology, Mbarara, Uganda; 37 Isotope Bioscience Laboratory–ISOFYS, Ghent University, Ghent, Belgium; 38 Integrated Center for Research, Development and Innovation in Advanced Materials, Nanotechnologies, and Distributed Systems for Fabrication and Control (MANSiD), Stefan cel Mare University of Suceava, Suceava, Romania; 39 Department of Forest Sciences, Luiz de Queiroz College of Agriculture, University of São Paulo, Piracicaba, Brazil; 40 Bavarian State Institute of Forestry, Freising, Germany; 41 Spatial Ecology and Conservation Laboratory, School of Forest Resources and Conservation, University of Florida, Gainesville, FL USA; 42 Institute of Biology, Geobotany and Botanical Garden, Martin Luther University Halle-Wittenberg, Halle-Wittenberg, Germany; 43 German Centre for Integrative Biodiversity Research (iDiv) Halle-Jena-Leipzig, Leipzig, Germany; 44 Department of Agriculture, Food, Environment and Forest (DAGRI), University of Firenze, Florence, Italy; 45 Department of Biological, Geological, and Environmental Sciences, University of Bologna, Bologna, Italy; 46 Department of Spatial Regulation, GIS and Forest Policy, Institute of Forestry, Belgrade, Serbia; 47 Department of Ecology and Evolutionary Biology, University of Connecticut, Storrs, CT USA; 48 Forest Research Institute, University of the Sunshine Coast, Sippy Downs, Queensland Australia; 49 Faculty of Natural Resources Management, Lakehead University, Thunder Bay, Ontario Canada; 50 Division of Forest Resources Information, Korea Forest Promotion Institute, Seoul, South Korea; 51 IFER–Institute of Forest Ecosystem Research, Jilove u Prahy, Czech Republic; 52 Global Change Research Institute CAS, Brno, Czech Republic; 53 Nicholas School of the Environment, Duke University, Durham, NC USA; 54 Department of Biology, University of Missouri-St Louis, St Louis, MO USA; 55 Programa de Pós-graduação em Biologia Vegetal, Instituto de Biologia, Universidade Estadual de Campinas, Campinas, Brazil; 56 Department of Plant Sciences and Conservation Research Institute, University of Cambridge, Cambridge, UK; 57 Andes to Amazon Biodiversity Program, Madre de Dios, Peru; 58 Facultad de Ciencias Forestales y Ambientales, Universidad Juárez del Estado de Durango, Durango, Mexico; 59 Department of Biology, West Virginia University, Morgantown, WV USA; 60 Department of Physical and Biological Sciences, The College of Saint Rose, Albany, NY USA; 61 Biology Department, Centre for Structural and Functional Genomics, Concordia University, Montreal, Quebec Canada; 62 Natural Science Department, Universidade Regional de Blumenau, Blumenau, Brazil; 63 World Agroforestry (ICRAF), Nairobi, Kenya; 64 Cirad, UMR EcoFoG (AgroParisTech, CNRS, INRAE), Université des Antilles, Université de la Guyane, Campus Agronomique, Kourou, France; 65 Department of Geographical Sciences, University of Maryland, College Park, MD USA; 66 Institute of Forestry, Belgrade, Serbia; 67 Institute of Botany, The Czech Academy of Sciences, Třeboň, Czech Republic; 68 Department of Botany, Faculty of Science, University of South Bohemia, České Budějovice, Czech Republic; 69 IRET, Herbier National du Gabon (CENAREST), Libreville, Gabon; 70 Department of Ecology and Evolutionary Biology, University of Arizona, Tucson, AZ USA; 71 The Santa Fe Institute, Santa Fe, NM USA; 72 Queensland Herbarium, Department of Environment and Science, Toowong, Queensland Australia; 73 Ecole de Foresterie et Ingénierie du Bois, Université Nationale d’Agriculture, Kétou, Benin; 74 School of Biological and Behavioural Sciences, Queen Mary University of London, London, UK; 75 Biology Centre of the Czech Academy of Sciences, Institute of Entomology, Ceske Budejovice, Czech Republic; 76 Geography, College of Life and Environmental Sciences, University of Exeter, Exeter, UK; 77 Museu Paraense Emílio Goeldi. Coordenação de Ciências da Terra e Ecologia, Belém, Pará Brazil; 78 Institute of Plant Sciences, University of Bern, Bern, Switzerland; 79 Forest Research Institute Malaysia, Kuala Lumpur, Malaysia; 80 Research and Innovation Center, Fondazione Edmund Mach, San Michele All’adige, Italy; 81 Forestry Division, Food and Agriculture Organization of the United Nations, Rome, Italy; 82 Glick Designs LLC, Hadley, MA USA; 83 Royal Botanic Garden Edinburgh, Edinburgh, UK; 84 Department of Plant Sciences, University of Oxford, Oxford, UK; 85 Department of Plant Systematics, University of Bayreuth, Bayreuth, Germany; 86 Cirad, UPR Forêts et Sociétés, University of Montpellier, Montpellier, France; 87 Department of Forestry and Environment, National Polytechnic Institute (INP-HB), Yamoussoukro, Côte d’Ivoire; 88 Tropical Forests and People Research Centre, University of the Sunshine Coast, Maroochydore, Queensland Australia; 89 Centre for Conservation Science, The Royal Society for the Protection of Birds, Sandy, UK; 90 Wild Chimpanzee Foundation, Liberia Office, Monrovia, Liberia; 91 Instituto de Investigaciones de la Amazonía Peruana, Iquitos, Peru; 92 Centre for Invasion Biology, Department of Mathematical Sciences, Stellenbosch University, Stellenbosch, South Africa; 93 Theoretical Ecology Unit, African Institute for Mathematical Sciences, Cape Town, South Africa; 94 National Institute of Amazonian Research, Manaus, Brazil; 95 Department of Forest Science, Tokyo University of Agriculture, Tokyo, Japan; 96 Institute of Dendrology, Polish Academy of Sciences, Kórnik, Poland; 97 Poznań University of Life Sciences, Department of Game Management and Forest Protection, Poznań, Poland; 98 Faculty of Biology, Białowieża Geobotanical Station, University of Warsaw, Białowieża, Poland; 99 Department of Geosciences and Natural Resource Management, University of Copenhagen, Copenhagen, Denmark; 100 Department of Plant Biology, Institute of Biology, University of Campinas, UNICAMP, Campinas, Brazil; 101 School of Biological Sciences, University of Bristol, Bristol, UK; 102 Forestry Faculty, Bauman Moscow State Technical University, Mytischi, Russia; 103 Field Museum of Natural History, Chicago, IL USA; 104 CAVElab-Computational and Applied Vegetation Ecology, Department of Environment, Ghent University, Ghent, Belgium; 105 CTFS-ForestGEO, Smithsonian Tropical Research Institute, Balboa, Panama; 106 Department of Physical and Environmental Sciences, Colorado Mesa University, Grand Junction, CO USA; 107 UniSA STEM and Future Industries Institute, University of South Australia, Adelaide, South Australia Australia; 108 Department of Botany, Dr Harisingh Gour Vishwavidyalaya (A Central University), Sagar, India; 109 Department of Agriculture, Forestry and Bioresources, Seoul National University, Seoul, South Korea; 110 Interdisciplinary Program in Agricultural and Forest Meteorology, Seoul National University, Seoul, South Korea; 111 National Center for Agro Meteorology, Seoul, South Korea; 112 Research Institute for Agriculture and Life Sciences, Seoul National University, Seoul, South Korea; 113 Graduate School of Agriculture, Kyoto University, Kyoto, Japan; 114 Institute for World Forestry, University of Hamburg, Hamburg, Germany; 115 Institute of Forestry and Engineering, Estonian University of Life Sciences, Tartu, Estonia; 116 Biodiversity and Natural Resources Program, International Institute for Applied Systems Analysis (IIASA), Laxenburg, Austria; 117 Department of Geography, University College London, London, UK; 118 Faculty of Forestry, Qingdao Agricultural University, Qingdao, China; 119 Center for Forest Ecology and Productivity, Russian Academy of Sciences, Moscow, Russia; 120 Environmental Change Institute, School of Geography and the Environment, University of Oxford, Oxford, UK; 121 AgroParisTech, UMR-AMAP, Cirad, CNRS, INRA, IRD, Université de Montpellier, Montpellier, France; 122 Departamento de Ciências Biológicas, Universidade do Estado de Mato Grosso, Nova Xavantina, Brazil; 123 Department of Environment and Geography, University of York, York, UK; 124 Flamingo Land, Malton, UK; 125 Department of Wildlife Management, College of African Wildlife Management, Mweka, Tanzania; 126 Departamento de Ecología y Recursos Naturales, Facultad de Ciencias, Universidad Nacional Autónoma de México, Mexico City, Mexico; 127 Universidad del Tolima, Ibagué, Colombia; 128 Colegio de Profesionales Forestales de Cochabamba, Cochabamba, Bolivia; 129 Jardín Botánico de Missouri, Pasco, Peru; 130 Universidad Nacional de San Antonio Abad del Cusco, Cusco, Peru; 131 Department of Environment and Development Studies, United International University, Dhaka, Bangladesh; 132 Laboratorio de geomática, Instituto de Silvicultura e Industria de la Madera, Universidad Juárez del Estado de Durango, Durango, Mexico; 133 Programa de doctorado en Ingeniería para el desarrollo rural y civil, Escuela de Doctorado Internacional de la Universidad de Santiago de Compostela, Santiago de Compostela, Spain; 134 Department of Environment and Development Studies, United International University, Dhaka, Bangladesh; 135 Universidad Estatal Amazónica, Puyo, Pastaza Ecuador; 136 Department of Evolutionary Biology and Environmental Studies, University of Zürich, Zurich, Switzerland; 137 Climate, Fire, and Carbon Cycle Sciences, USDA Forest Service, Durham, NC USA; 138 Centre for Forest Research, Université du Québec à Montréal, Montreal, Quebec Canada; 139 V. N. Sukachev Institute of Forest, FRC KSC, Siberian Branch of the Russian Academy of Sciences, Krasnoyarsk, Russia; 140 Forest Ecology and Forest Management Group, Wageningen University and Research, Wageningen, The Netherlands; 141 Department of Ecology and Environmental Sciences, Pondicherry University, Puducherry, India; 142 Instituto Nacional de Tecnología Agropecuaria (INTA), Universidad Nacional de la Patagonia Austral (UNPA), Consejo Nacional de Investigaciones Científicas y Tecnicas (CONICET), Río Gallegos, Argentina; 143 School of Social Sciences (Urban Studies), Western Sydney University, Penrith, New South Wales Australia; 144 Forestry Department, Food and Agriculture Organization of the United Nations, Rome, Italy; 145 Instituto Nacional de Pesquisas da Amazônia, Manaus, Brazil; 146 Laboratório de Dendrologia e Silvicultura Tropical, Centro de Formação em Ciências Agroflorestais, Universidade Federal do Sul da Bahia, Itabuna, Brazil; 147 Jardín Botánico de Medellín, Medellin, Colombia; 148 Chair for Forest Growth and Yield Science, TUM School for Life Sciences, Technical University of Munich, Munich, Germany; 149 Universidad Nacional de la Amazonía Peruana, Iquitos, Peru; 150 Servicios Ecosistémicos y Cambio Climático (SECC), Fundación Con Vida & Corporación COL-TREE, Medellín, Colombia; 151 Centro Agricoltura, Alimenti, Ambiente, University of Trento, San Michele All’adige, Italy; 152 Department of Biological Sciences, Boise State University, Boise, ID USA; 153 Department of Biology, University of Florence, Florence, Italy; 154 Tropical Biodiversity, MUSE–Museo delle Scienze, Trento, Italy; 155 Info Flora, Geneva, Switzerland; 156 Department of Environmental Sciences, Central University of Jharkhand, Ranchi, Jharkhand India; 157 Centro de Modelación y Monitoreo de Ecosistemas, Universidad Mayor, Santiago, Chile; 158 Vicerrectoria de Investigacion y Postgrado, Universidad de La Frontera, Temuco, Chile; 159 Depto. de Silvicultura y Conservacion de la Naturaleza, Universidad de Chile, Temuco, Chile; 160 Datascientist.ch, Wallisellen, Switzerland; 161 Siberian Federal University, Krasnoyarsk Russian Federation, Krasnoyarsk, Russia; 162 Geobotany, Faculty of Biology, University of Freiburg, Freiburg im Breisgau, Germany; 163 National Forest Centre, Forest Research Institute Zvolen, Zvolen, Slovakia; 164 Université de Lorraine, AgroParisTech, INRAE, Silva, Nancy, France; 165 Center for Ecological Dynamics in a Novel Biosphere (ECONOVO) and Center for Biodiversity Dynamics in a Changing World (BIOCHANGE), Department of Biology, Aarhus University, Aarhus, Denmark; 166 Forest Ecology and Forest Management, Wageningen University and Research, Wageningen, The Netherlands; 167 Faculty of Environmental Sciences and Natural Resource Management, Norwegian University of Life Sciences, Ås, Norway; 168 Departamento de Biología, Universidad de la Serena, La Serena, Chile; 169 Centro de Ciências Biológicas e da Natureza, Universidade Federal do Acre, Rio Branco, Acre Brazil; 170 Guyana Forestry Commission, Georgetown, France; 171 Environmental and Life Sciences, Faculty of Science, Universiti Brunei Darussalam, Bandar Seri Begawan, Brunei; 172 Plant Systematic and Ecology Laboratory, Department of Biology, Higher Teachers’ Training College, University of Yaoundé I, Yaoundé, Cameroon; 173 Departamento de Ecologia, Universidade Federal do Rio Grande do Norte, Natal, Rio Grande do Norte Brazil; 174 Warsaw University of Life Sciences, Warsaw, Poland; 175 Section for Ecoinformatics & Biodiversity, Department of Biology, Aarhus University, Aarhus, Denmark; 176 Faculty of Forestry and Wood Sciences, Czech University of Life Sciences, Prague, Czech Republic; 177 Wildlife Conservation Society, Vientiane, Laos; 178 Quantitative Biodiversity Dynamics, Betafaculty, Utrecht University, Utrecht, The Netherlands; 179 Iwokrama International Centre for Rainforest Conservation and Development (IIC), Georgetown, Guyana; 180 School of Forestry and Environmental Studies, Yale University, New Haven, CT USA; 181 Botanical Garden of Ural Branch of Russian Academy of Sciences, Ural State Forest Engineering University, Yekaterinburg, Russia; 182 Pontificia Universidad Católica del Ecuador, Quito, Ecuador; 183 LINCGlobal, Museo Nacional de Ciencias Naturales, CSIC, Madrid, Spain; 184 Plant Ecology and Nature Conservation Group, Wageningen University, Wageningen, The Netherlands; 185 Silviculture Research Institute, Vietnamese Academy of Forest Sciences, Hanoi, Vietnam; 186 Department of Biology, Stanford University, Stanford, CA USA; 187 Centre for the Research and Technology of Agro-Environmental and Biological Sciences, CITAB, University of Trás-os-Montes and Alto Douro, UTAD, Viseu, Portugal; 188 Department of Ecology and Sustainable Agriculture, Agricultural High School, Polytechnic Institute of Viseu, Viseu, Portugal; 189 Department of Forest Engineering Universidade Regional de Blumenau, Blumenau, Brazil; 190 Environmental Studies and Research Center, University of Campinas, UNICAMP, Campinas, Brazil; 191 Department of Forest and Wood Science, University of Stellenbosch, Stellenbosch, South Africa; 192 Key Laboratory of Tropical Biological Resources, Ministry of Education, School of Life and Pharmaceutical Sciences, Hainan University, Haikou, China; 193 Division of Forestry and Natural Resources, West Virginia University, Morgantown, WV USA; 194 Department of Zoology, University of Oxford, Oxford, UK; 195 Manaaki Whenua–Landcare Research, Lincoln, New Zealand; 196 Department of Wetland Ecology, Institute for Geography and Geoecology, Karlsruhe Institute for Technology, Karlsruhe, Germany; 197 Independent Researcher, Bad Aussee, Austria; 198 Centre for Agricultural Research in Suriname (CELOS), Paramaribo, Suriname; 199 Tropenbos International, Wageningen, The Netherlands; 200 Polish State Forests, Coordination Center for Environmental Projects, Warsaw, Poland; 201 Research Center of Forest Management Engineering of State Forestry and Grassland Administration, Beijing Forestry University, Beijing, China; 202 Department of Genetics, Evolution, and Environment, University College London, London, UK

**Keywords:** Invasive species, Biogeography, Forest ecology

## Abstract

Determining the drivers of non-native plant invasions is critical for managing native ecosystems and limiting the spread of invasive species^1,2^. Tree invasions in particular have been relatively overlooked, even though they have the potential to transform ecosystems and economies^3,4^. Here, leveraging global tree databases^5–7^, we explore how the phylogenetic and functional diversity of native tree communities, human pressure and the environment influence the establishment of non-native tree species and the subsequent invasion severity. We find that anthropogenic factors are key to predicting whether a location is invaded, but that invasion severity is underpinned by native diversity, with higher diversity predicting lower invasion severity. Temperature and precipitation emerge as strong predictors of invasion strategy, with non-native species invading successfully when they are similar to the native community in cold or dry extremes. Yet, despite the influence of these ecological forces in determining invasion strategy, we find evidence that these patterns can be obscured by human activity, with lower ecological signal in areas with higher proximity to shipping ports. Our global perspective of non-native tree invasion highlights that human drivers influence non-native tree presence, and that native phylogenetic and functional diversity have a critical role in the establishment and spread of subsequent invasions.

## Abstract (web summary)

Analysis combining multiple global tree databases reveals that whether a location is invaded by non-native tree species depends on anthropogenic factors, but the severity of the invasion depends on the native species diversity.

## Main

Plant invasions have multifaceted impacts on ecosystems and human wellbeing across the globe^1–3,8^. It is expected that plant invasions will continue to increase in the coming decades owing to human-assisted introduction and naturalization of these species, with ever-growing impacts on biodiversity within native forest ecosystems^1,9,10^. These invasions will undoubtedly also have considerable economic impacts in managed landscapes by disrupting timber production, agriculture and human livelihoods^11–17^. In particular, non-native trees represent an important and increasing concern globally, as they are often actively planted far outside their native ranges for forestry, reforestation, residential, or ornamental purposes^4,18^. Along with the passive spread of non-native species, the active propagation of trees by humans can often result in an increased potential to become problematic invaders^4,19–21^. Given the prominent roles of trees in shaping the structure and functioning of ecosystems, such tree invasions have the capacity to alter plant composition, productivity, biodiversity and the services provided to humans^1,4,22^. Previous research in invasion ecology has expanded our understanding of community-level properties that influence ecosystem susceptibility to invasion^23–25^, as well as traits that make plant species more likely to become invasive^26–30^. However, most work has been restricted to local and regional scales^31,32^, with contrasting ecological mechanisms affecting invasion success in different regions. We thus lack a global unified theory of the human and ecological drivers of tree species invasions^33^. Developing an integrated global understanding of ecological and anthropogenic forces that drive non-native tree invasions is critical to improve decision making in conservation and management.

Countless ecological mechanisms have been proposed to explain the susceptibility of different ecosystems to invasion by non-native species in different locations. Traditionally, more diverse or ecologically complex systems are thought to exhibit ‘biotic resistance’ to invasion^23,34–39^. This hypothesis is based on the assumption that greater diversity in the native community fills the available ecological niches and reduces available resources, limiting niche space to novel species. However, most work has focused on testing this hypothesis using species richness as an indicator of niche filling^23,35^, which may not fully capture the proportion of niches that are filled in the native community. Instead, more informative metrics for niche filling may be phylogenetic or functional diversity. Phylogenetic diversity accounts for evolutionary similarity and represents a reasonable proxy for similarity between taxa, whereas functional diversity directly addresses the underlying mechanism of biotic resistance (that is, the breadth of ecological niches filled), but may be more difficult to measure. Conversely, there is also evidence for the opposite pattern in some ecosystems, whereby a more diverse community is indicative of a more favourable habitat, where a wide range of invasive species might survive. This ‘biotic acceptance’^25,40,41^ hypothesis leads to the expectation that highly diverse sites are optimal for many plant species and could promote invasion of non-native species. Nonetheless, we still lack a unified understanding of the relative importance of these two competing processes, and their variation across the globe, leading to ongoing calls to resolve this ‘invasion paradox’^25^.

Invasion success is also likely to depend on the ecological strategy of the invading species relative to the recipient native community. One school of thought is that environmental constraints are the primary drivers of plant species distributions. Therefore, to be successful, invasive species ought to be similar to native species that are adapted for that region, especially in extreme environments^42^. Under this ‘environmental filtering hypothesis’^43,44^ (or ‘preadaptation hypothesis’), invasive species will be more successful if their traits mirror those of the native community^45^. For example, to be successful in a harsh desert environment, non-native plants would need to be ecologically similar to native plants to survive, possessing traits that protect them against high heat and water loss. By contrast, the ‘limiting similarity hypothesis’ (also known as ‘Darwin’s naturalization hypothesis’) postulates that invasive species need to be ecologically distinct from native species to avoid niche overlap^46–49^. Here, invaders are thought to be more successful if they can fill unique niche spaces that are not already used by the native community, reducing competition and enabling their establishment. These two processes suggest contrasting mechanisms for how species invade: either species invade by being similar or dissimilar to the native community (Darwin’s naturalization conundrum^24,50^). It is possible that the relative importance of these opposing ecological mechanisms varies under different environmental conditions, with greater importance of environmental filtering in harsh conditions and greater niche differentiation in more moderate environments^51,52^. Such regional variation in the relative importance of these mechanisms might help to explain the opposing responses observed across studies. However, until now, we lack a broad-scale analysis of these different invasion mechanisms that can help us to see past the idiosyncrasy of local-scale observations to identify unifying trends.

A key challenge hindering a global consensus of the ecological patterns and mechanisms underpinning plant invasion is that these processes are likely strongly influenced by anthropogenic activity, which may dampen the signal of ecological drivers. Humans drive contemporary plant invasions through highly efficient transport—both intentional and accidental—of non-native plants, with proximity to ports and airports being associated with increased invasion^11,53,54^. A constant influx of non-native species may override a native community’s ability to resist invasion^55^ (biotic resistance) and obscure the impacts and importance of specific ecological drivers, such as native diversity, particularly at early stages of invasion. That is, with increased propagule pressure of non-natives species exerted by humans, the relative importance of ecological drivers may be reduced. Moreover, sites with high levels of non-native propagule pressure due to human activity are also likely to be heavily disturbed, compounding this anthropogenic influence. Accounting for human global change drivers may be particularly important when considering the role of invasion strategy, with the potential for anthropogenic drivers and human propagule pressure to overwhelm the impact of ecological drivers. This could occur through an increase in the frequency and magnitude of introductions, which would be expected to increase stochastic variation and dampen ecological signals. So far, these hypotheses have been tested only at local and regional scales, with few studies integrating ecological and anthropogenic drivers of invasion at the global scale to disentangle the relative importance of human activity, environmental conditions and biological diversity^33^.

Here, by combining global datasets of local-scale forest inventories, native status, environmental climate variables and anthropogenic drivers, we test for the relative importance of ecological and anthropogenic influence on non-native tree invasion. Using this large-scale approach, we search for a unifying perspective of the environmental and anthropogenic contexts driving non-native invasion and invasion severity, via both relative richness and abundance of non-natives, as well as invasion strategy. We consider three hypotheses: (H1) greater native diversity reduces non-native invasion^23^; (H2) high levels of environmental filtering in extreme environmental conditions leads to similarity of non-natives with the surrounding natives, and moderate conditions are associated with greater levels of niche differentiation and dissimilarity^24^; and (H3) human drivers, specifically proximity to ports and areas of high human population density, will mediate and potentially override these ecological relationships^56^. We explore these hypotheses through the lens of different biodiversity metrics (phylogenetic diversity, functional diversity and species richness), providing a comprehensive view of the interactions between ecological processes and human influence on invasion. Addressing these hypotheses is important to highlight generalizations in the field for prevention and management of non-native tree invasions, which is key to mitigating the potential severe ecological and socio-economic toll of these invasions.

**Fig. 1:**
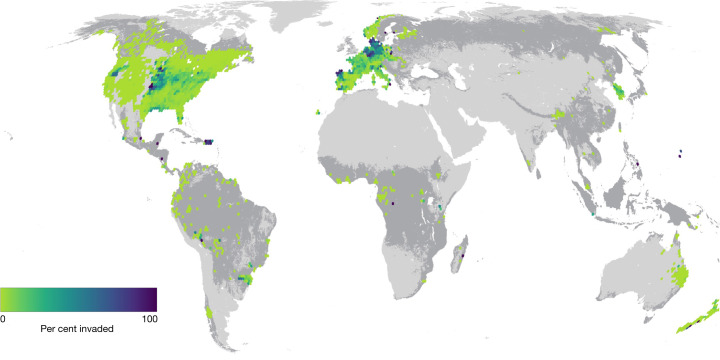
Distribution of the study data. Distribution of the full study dataset, coded for non-native severity (*n* = 471,888 plots). The map shows average per cent invasion across a 1-degree hexagonal grid, from non-invaded (0%) pixels in green to completely invaded (100%) pixels in purple. Plots are considered invaded if there is any non-native tree present.

**Extended Data Fig. 1:**
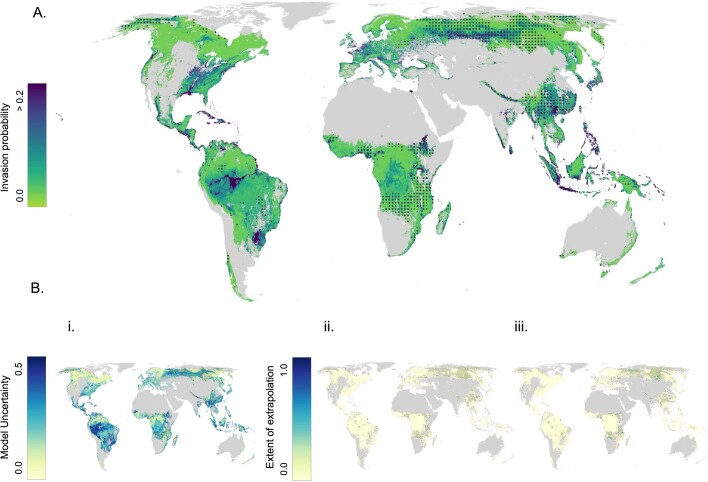
Map of non-native invasion probability. Map showing probability of non-native tree presence based on the probability output of the random forest classifier (A, total n = 368,030 plots, n per iteration = 10,000 plots) alongside maps showing uncertainty in predictions (B) including local uncertainty of invasion probability via bootstrapped coefficient of variation (i) and extent of extrapolation as percentage of bands outside univariate (ii) and multivariate (ii) training range. Regions outside the Area of Applicability are indicated with dots.

Using the Global Forest Biodiversity Initiative database^7^, we determined native tree status (native or non-native) according to the Global Naturalized Alien Flora^6^ and the KEW Plants of the World databases^5^. This dataset encompassed 471,888 plots, of which 4.9% of plots were invaded, or contained at least one non-native tree species (Fig. 1 and Supplementary Table 1a). Moreover, this dataset contained a larger proportion of invaded plots in tropical (15.2%) than in temperate systems (5.2 %). Overall, 249 individual non-native tree species were identified, with the most frequent being *Robinia pseudoacacia*, *Pinus sylvestris*, *Maclura pomifera*, *Picea abies* and *Ailanthus altissima* labelled as non-native in 3,976, 2,603, 2,493, 2,468 and 1,597 plots, respectively (Supplementary Table 2). Regions with the greatest likelihood of being invaded include North America, Europe and East Asia (Extended Data Fig. 1), consistent with previous findings^10,57^ (but see ref. ^58^). To test for drivers of non-native tree invasion and invasion strategy, we used a down-sampled version of the dataset consisting of 17,738 forest plots, distributed across 14 biomes proportional to their global land cover.

We calculated three metrics of invasion: (1) presence of non-natives in the plot (‘non-native presence’); (2) relative proportion of non-native species richness to total tree richness (‘non-native richness’); and (3) relative proportion of non-native species basal area to total tree basal area (‘non-native abundance’). The first metric (non-native presence) is simply a measure of the presence or absence of invasion, whereas the latter two metrics (relative abundance and richness) provide insight into the subsequent severity of the invasion.

To test how hypothesized human and environmental drivers affected the probability a forest plot was invaded or the invasion severity within invaded plots, we built generalized linear models (GLMs) and random forest models using either phylogenetic or functional diversity metrics (both as richness and redundancy) as predictor variables (Extended Data Fig. 3). For both functional and phylogenetic diversity, we used random forest models to determine variable importance and for visualization purposes, whereas GLMs were used to test for significance and directionality of relationships. Our models also included human drivers (distance to shipping ports (hereafter referred to as ports) and population density) and accounted for several additional soil chemical and climate variables. Next, to test whether non-native tree species invade by being similar or dissimilar to the native community (termed ‘invasion strategy’), we again built models predicting non-native similarity from either native phylogenetic or functional diversity metrics, along with the same environmental and human impact variables. The non-native invasion strategy was defined as the change in redundancy due to addition of non-native trees, with values below zero and values above zero indicating invasion via similarity and dissimilarity, respectively, to the native community.

## Diversity limits invasion severity

**Fig. 2:**
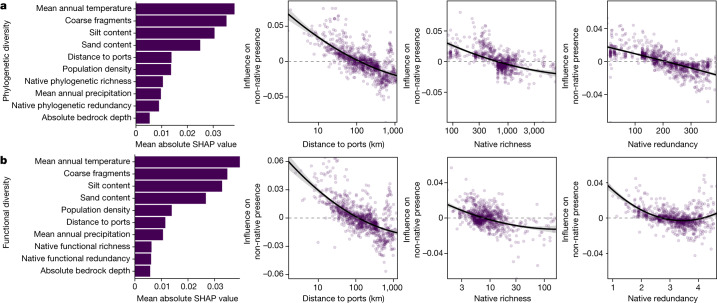
Anthropogenic drivers are more important than native diversity in determining invasion occurrence. **a**,**b**, Importance (Shapley additive explanations (SHAP) values) of all variables included in random forest models ordered from greatest to least important, alongside influence of distance to ports, native richness and native redundancy on non-native presence (whether a plot is invaded or not) for global models of phylogenetic (**a**) and functional (**b**) diversity (phylogenetic diversity, *n* = 17,640 plots; functional diversity, *n* = 17,271 plots). All results shown are from random forest models. Note that *y*-axis ranges differ among panels, with the variable importance plots representing the corresponding magnitude. Error bands represent 95% confidence intervals.

We found that anthropogenic drivers were more important than local native tree diversity in determining non-native invasion (presence) globally (H3), whereas native diversity— both phylogenetic and functional—was most important in determining invasion severity (H1; Fig. 2 and Supplementary Tables 3 and 4; phylogenetic diversity random forest area under the curve (AUC) = 0.634, functional diversity random forest AUC = 0.631). These results indicate the importance of human-induced propagule pressure in initiating invasion of forests and of native biodiversity moderating the severity of the invasion. We found that forest plots closer to ports are more likely to be invaded (Supplementary Tables 3 and 4; linear model *P* < 0.001). Notably, these results are consistent whether we analyse all data together at the global level or separate data into either the temperate and tropical bioclimatic zones (Supplementary Tables 3 and 4). By contrast, we did not find that human population density was consistently related to non-native presence, with results being variable across diversity metrics and bioclimatic zones considered (Supplementary Tables 3 and 4). However, population density was always positively correlated with invasion probability; population density may be a weaker predictor as it only measures human presence, which is not necessarily related to propagule pressure.

**Fig. 3:**
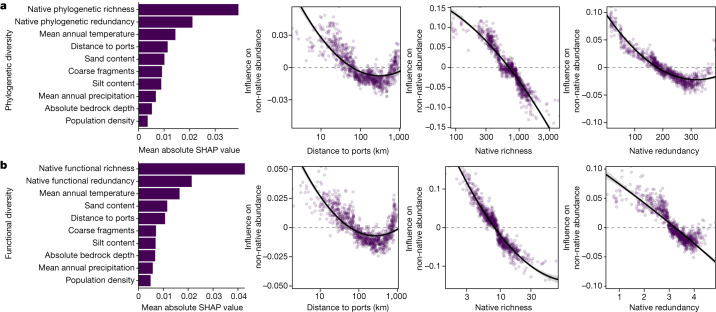
Native diversity is the most important driver of invasion severity. **a**,**b**, Importance (Shapley additive explanations (SHAP) values) of all variables included in random forest models ordered from greatest to least important, alongside influence of distance to ports, native richness and native redundancy on invasion severity for global models of phylogenetic (**a**) and functional (**b**) diversity (phylogenetic diversity, *n* = 3,498 plots; functional diversity, *n* = 3,368 plots). Plots are shown for the severity of invasion measured as non-native species abundance (proportion of basal area with non-native plant species); plots for non-native species richness (proportion of non-native plant species) are shown in Extended Data Fig. 4. All results shown are from random forest models. Note that the *y*-axis ranges differ among panels, with the variable importance plots representing the corresponding magnitude. Error bands represent 95% confidence intervals.

Proximity to ports has long been known to influence invasion^11,53,54^, with locations closer to a port being likely to experience greater propagule pressure. Moreover, proximity to ports may serve as a proxy for residence time, where plots closer to ports are more likely to have longer exposure to non-native propagule pressure, thus increasing the likelihood of invasion^56^. Yet, at far enough distances, stochastic processes and historical land-use patterns may begin to weaken the role of ports (Fig. 3, distances greater than 500 km). For example, the third most frequent non-native tree in our dataset, *M. pomifera*, is widely naturalized throughout the interior of North America, where it has been used for various agricultural purposes dating back to the 1850s^59^. Such results highlight the idiosyncratic use of trees across the globe, leading to unique invasion trends relative to herbaceous plants. Nevertheless, at more local scales, this strong signal of anthropogenic activity and associated propagule pressure relative to native diversity driving non-native presence is in agreement with previous work that considers invasion across stages^56^ and recent assessments of regional and global tree invasion^57,60^, and highlights the prominent role of humans in reshaping biological communities.

Although proximity to ports determined the probability a forest plot was invaded, native tree communities with higher phylogenetic and functional diversity exhibited lower invasion severity (Fig. 3, Extended Data Fig. 4 and Supplementary Tables 3 and 4; phylogenetic diversity random forest non-native richness *R*^2^ = 0.68, phylogenetic diversity random forest non-native abundance *R*^2^ = 0.14, functional diversity random forest non-native richness *R*^2^ = 0.69 and functional diversity random forest non-native abundance *R*^2^ = 0.07; GLM phylogenetic and functional diversity *P* < 0.001). Additionally, distance to ports was no longer significant in linear models predicting invasion severity (Supplementary Tables 3 and 4) for both phylogenetic (*P* = 0.16 and 0.28 for non-native richness and abundance, respectively) and functional diversity models (*P* = 0.63 and 0.86 for non-native richness and abundance, respectively), and showed reduced variable importance in the random forest models (Fig. 3 and Extended Data Fig. 4). When investigating these patterns using conventionally analysed species richness instead of phylogenetic or functional richness, we find similar qualitative results (Supplementary Table 5, random forest non-native richness *R*^2^ = 0.71 and random forest non-native abundance *R*^2^ = 0.14), suggesting that species diversity may be a useful proxy for projecting invasion severity in the absence of functional and phylogenetic information. Our results are consistent with the hypothesis of biotic resistance (H1), where increased native diversity reduces invasion success, which is probably driven by the native community utilizing more available niche spaces^23,34–36,61^. These results are also consistent with work investigating tree migration drivers that suggests that migration is slower into more diverse communities owing to greater resource use (fewer available niches) in these systems^57^.

Overall, these results show that anthropogenic drivers, particularly distance to shipping centres (ports), are more important in determining which locations will experience non-native invasions compared with traditionally studied native diversity (H3). However, it is the intrinsic ecological drivers, including native tree community phylogenetic and functional diversity (richness and redundancy), that are more important in determining invasion severity (H1). Repeated human introduction of plant species has a more important role in the initial invasion process, but invasion severity is predominantly a result of native intrinsic diversity. Notably, both distance to ports and native diversity show patterns of saturation of effects, suggesting a threshold at which plots that are far enough from ports, or high enough in native diversity, will not benefit from further distance or diversity with regard to reduced invasion or invasion severity. Although our focus here is on the relative importance of human versus biotic drivers of introduction, we find that environmental variables—especially mean annual temperature—correlate strongly with patterns of non-native invasion, which may reflect resource availability^26^, belowground microorganism composition^30^ or potential climate compatibility between donor and recipient ranges^62^. Together, our results suggest that locations near human activity are more likely to experience non-native invasions in part due to increased propagule pressure, whereas those with lower diversity are more likely to experience more severe non-native invasions once non-natives are present. These results may suggest that managing forests to maintain high native tree diversity may be a good strategy to buffer communities against invasion, particularly for locations that are far from human activity.

## Evidence for environmental filtering

**Fig. 4:**
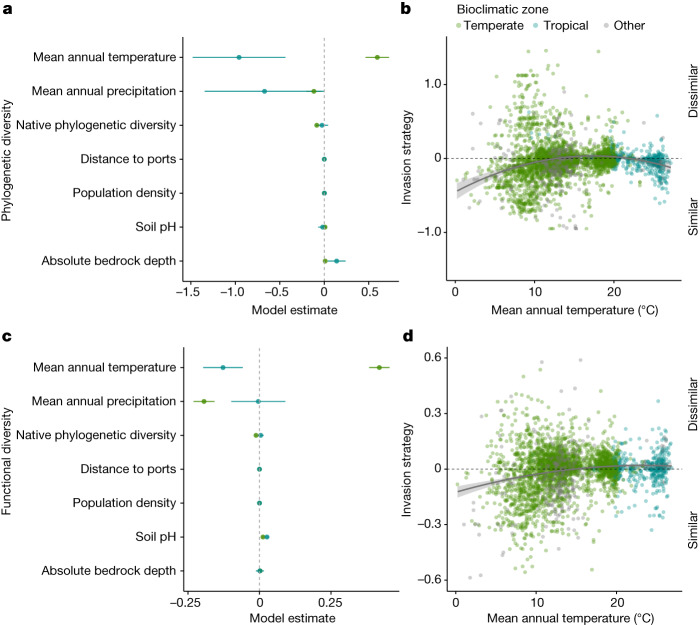
Environmental filtering at temperature extremes. **a**,**c**, Estimates of overlapping variables included in temperate and tropical GLM models (forest plot) for phylogenetic (**a**) and functional (**c**) diversity models (phylogenetic diversity, *n* = 3,498; functional diversity, *n* = 3,368). Values to the left of the zero line indicate negative model estimates, and those to the right indicate positive estimates. **b**,**d**, Relationship between mean annual temperature and invasion strategy for phylogenetic (**b**) and functional (**d**) diversity models, showing that at extreme temperatures invasion occurs through similarity (Supplementary Table 7; phylogenetic diversity: *P*_(1)_ = 9.69 × 10^−14^, *P*_(2)_ = 2.13 × 10^−11^; functional diversity: *P*_(1)_ < 2 × 10^−16^, *P*_(2)_ = 1.07 × 10^−4^, where *P*_(1)_ and *P*_(2)_ represent each temperature and temperature squared *P* values, respectively). Note for functional diversity, this pattern only holds at low temperatures. Error bars and bands represent standard error.

**Fig. 5:**
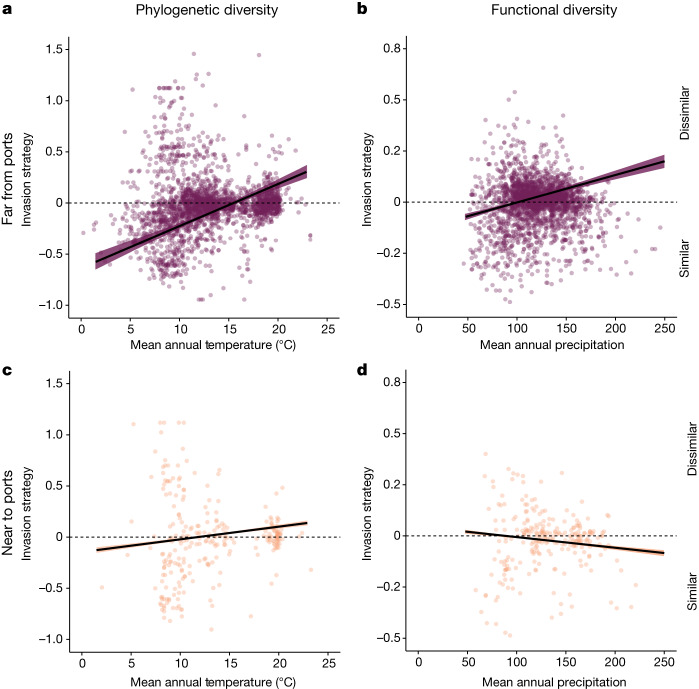
Proximity to ports weakens environmental filtering in the temperate bioclimate zone. **a**,**b**, In temperate plots far from ports, temperature is positively correlated with an invasion strategy of increasing dissimilarity for phylogenetic (**a**) and functional (**b**) diversity (phylogenetic diversity: *n* = 2,710 plots, *P* = 6.37 × 10^−6^; functional diversity: *n* = 2,603, *P* < 2 × 10^−16^). **c**,**d**, This relationship between temperature and invasion strategy weakens for phylogenetic (**c**) and functional (**d**) diversity with proximity to ports (Supplementary Table 7; phylogenetic diversity: *P* = 0.0001; functional diversity: *P* = 2.71 × 10^−13^). Lines and points represent the lowest (**c**,**d**) and highest (**a**,**b**) 10% of data. Error bands represent standard error.

When considering a range of climate, soil and anthropogenic variables, we find evidence for environmental filtering as a driver of invasion strategy, in particular, with respect to mean annual temperature and precipitation. In all global models, temperature was important for predicting tree invasion strategy (Fig. 4, Extended Data Fig. 5 and Supplementary Table 6; phylogenetic diversity random forest *R*^2^ = 0.084, functional diversity random forest *R*^2^ = 0.099; H2), with our global analysis indicating that non-native trees were more similar to the native community in environments at cold and hot temperature extremes (Fig. 5 and Supplementary Table 6, *P* < 0.001). That is, in order to invade into a cold or hot environment, non-native plants are more successful if they share similar traits with native plants to survive in these harsher temperature conditions. By contrast, at locations with moderate temperatures, non-natives are neither more nor less similar to native communities, potentially because these less harsh environmental conditions allow a wider range of life strategies to coexist^51^. For functional diversity, invasion strategy at high temperatures is relatively neutral, with the line approaching a value of zero, suggesting that although phylogenetically similar, these communities show some level of functional divergence, highlighting the importance of including functional diversity in future studies. When separating the data into temperate and tropical systems, we found divergent temperature patterns (Supplementary Table 6; temperate *P* < 0.001, tropical *P* = 0.01). In temperate systems, non-native trees were more likely to be similar to the native tree community in colder environments relative to hot environments, in line with previous results in temperate North America^63^. In tropical systems, we found the opposite pattern, with non-native trees being more likely to be similar to the native tree community in hotter tropical environments. At the lowest temperatures, non-natives invading through similarity were primarily gymnosperms (fir, spruce and pine species) invading into native communities containing species in the same genus; by contrast, at the highest temperatures, non-natives invading through similarity were angiosperms, with a high prevalence of palms and legumes. Further, we detect a similar pattern of environmental filtering for mean annual precipitation when analysing phylogenetic and functional diversity with random forest models, where lower or higher precipitation is associated with non-native invasion through similarity (Extended Data Fig. 5). This suggests that the most likely invaders at low or high temperature or precipitation may be ecologically similar to the host communities, which could inform invasion risk checklists at ports.

Within the temperate bioclimatic zone, we found evidence that anthropogenic activity weakened the environmental filtering pattern for phylogenetic and functional diversity seen for temperature and precipitation, respectively (H3). In particular, proximity to ports modified the signal of environmental filtering due to temperature, weakening the influence of temperature on invasion strategy with respect to phylogenetic similarity (Fig. 5 and Supplementary Table 6; *P* < 0.001). Colder ecosystems show evidence of environmental filtering of invasion; however, increased proximity to ports reduces the prevalence of this strategy. We suggest that this may be due to increased introductions around shipping ports, which would increase stochastic variation and dampen ecological strategies. However, we did not detect a similar interaction governing the tropical bioclimatic zone, potentially owing to relatively lower human pressure, and particularly lower ship traffic^64^, compared to temperate systems. Alternatively, this pattern may also reflect the fact that some temperate plots occur at greater distances to ports than tropical sites (95th percentile of 784 km versus 311 km for temperate and tropical, respectively), increasing statistical power for detecting this trend in temperate regions. Furthermore, proximity to ports also marginally weakened the signal of environmental filtering due to precipitation for functional invasion strategy (Supplementary Table 6; *P* = 0.07). These results illustrate that human influence can override the ecological factors driving invasion, suggesting that at high enough propagule pressure, the phylogenetic and functional similarity of a non-native becomes less important in predicting its ability to invade a native community. Nevertheless, as our analyses are not causal, these results could also reflect correlations between port locations and invasion strategy. However, when we investigated the same effect with human population density, we did not see this weakening effect, demonstrating that distance to ports seems to be a particularly relevant mediator of these patterns. These results suggest that human activity may overwhelm ecological drivers of non-native invasion strategies and reduce the influence of ecological processes, making inclusion of human impacts critical for studying global invasion strategies.

Collectively, our work integrates biotic and anthropogenic factors across phylogenetic and functional diversity for both invasion presence and invasion severity of non-native tree species worldwide. Although non-native trees have been relatively overlooked relative to herbaceous plants, their large size, long lifespans and important history in forestry, food, reforestation and city landscaping exposes trees to unique ecological and anthropogenic factors that shape their worldwide distributions. Moreover, given that many tree invasions are in their infancy, with substantial ‘invasion debts’ of recent tree plantings^3^, understanding the ecological drivers promoting spread has the potential to provide real-time feedback for the preventative management of invasive trees. However, there are important considerations when interpreting these findings, many of which could be addressed with increased data resolution and increased sampling within under-sampled geographic regions. First, our analysis is largely observational, whereas community composition would ideally be compared before and after invasion to better understand the causality of the trends observed here. We can gain some insight into this question by conducting a sensitivity analysis on the subset of invaded plots that were measured at multiple time points and that had no initial invasion. Doing so reveals that the reduction in native diversity due to invasion can potentially account for as much as 10.4% (mean of 6.7%) of the observed biotic resistance (Supplementary Table 9), but that the remainder of this effect is attributable to difference in native diversity (that is, biotic resistance) across plots. Additional long-term data on plots that are uninvaded and become invaded will be useful in further addressing the influence of invasion on native diversity. Second, many tree species in our analysis were only identified to genus level or were not present in the master plant phylogeny, which may lead to an underestimation of native diversity or invasive species richness in some plots, particularly in species-rich forests. Indeed, a key challenge in global analyses such as ours is the underrepresentation of certain ecosystems, for example, tropical ecosystems^58^. This is addressed to some extent by our down-sampling approach, as well as our spatial cross-validation approach (Methods), but ongoing efforts to fund and develop open-access and fair^65^ tropical forest inventory data are critical for gaining better insight into these ecologically and socially important ecosystems.

Many tree species are intentionally introduced for forestry or wood products and may be managed^4^, generating variation in the drivers underpinning invasion that are unique to trees. To minimize the influence of heavily managed forests, we included only plots with a minimum of three species and thus our dataset does not include monoculture forestry plantations. In addition, when restricting our analysis to the subset of global plots that occur in protected areas with minimal human footprint, our core results and inferences remain unchanged (Supplementary Table 7). Having additional high-quality data on the human role in invasion, including the type and time of management, and overall level in disturbance regime^66^, would refine our results and better separate ecological versus human drivers. Future work should also focus on drivers of tree invasion and invasion strategies across scales^25,63,67^, as patterns may differ at scales larger than the local plot level that we include here, which may be important for regional versus local management of non-native trees. Finally, emerging work shows that the consideration of native range size and change in environment and/or disturbance from donor to recipient community may be more helpful in understanding introduction and invasion success than simply quantifying these variables in the novel, recipient range^62,66^. Therefore, including the change in environmental and human impact variables would also be a fruitful avenue for future research.

Together, these results provide important unifying insights into the global drivers of non-native tree invasions and the ecological strategies that might be most successful in different regions. The trends and ecological mechanisms identified here can provide tangible guidelines to support forest management of non-native tree invasions around the globe. However, because non-native trees are introduced purposefully for forestry or to support local livelihoods, which can lead to differences in forest management objectives and strategies^4^, it is critical that local stakeholders are included when making decisions about how to best manage these introductions^68,69^. Ultimately, this emerging understanding of global tree invasions provides fundamental insights that are needed to understand how forest composition is being reshaped under global change, and for forest management practices to limit the spread and impacts of non-native tree invasions worldwide.

## Methods

### Tree inventory and non-native status

For tree inventory data, we used the Global Forest Biodiversity Initiative (GFBI) database^7^, which contains tree-level abundance data for more than 1.2 million forest plots on all continents across the globe, containing more than 31 million unique georeferenced records of tree size and density dating from 1958. Each observation in the dataset consists of a unique tree ID, plot ID, plot coordinates, tree diameter at breast height (DBH), tree-per-hectare expansion factors, year of measurement, and binomial species names. In this study, we applied several filters to these data before analyses. First, where plots had multiple years of data, we kept only the most recent year of census data. We then subset the data to include only plots with at least three species as required for our phylogenetic metrics, excluding monoculture forest plantations from the study.

To assign native status to each tree species (native or non-native, representing naturalized and invasive), we established a consensus status between the Global Naturalized Alien Flora (GloNAF)^6^ and the KEW Plants of the World^70^ databases. All databases were standardized to The Plant List taxonomy^71^. The GloNAF database contains detailed, georeferenced information on the naturalized status of more than 10,000 plant species in each of 1,029 regions across the globe representing countries or federal states; the KEW database outlines native ranges of vascular plant species for over 1.2 million plant species^70^. The GFBI and GloNAF datasets were joined by matching each unique species by location in GFBI to a GloNAF region polygon and species status. Then, for each GFBI plot, we extracted the GloNAF region identifier using Google Earth Engine^72^. This process was then repeated for the KEW database. We then filtered out plots that included any species with disagreement between GloNAF and KEW databases (that is, conflicting native status), and only included trees with a minimum diameter of 5 cm and a minimum height of 1.3 m to allow for DBH measurements. All trees identified as ‘non-native’ were verified to be listed in the BGCI Tree List, which defines a tree as, “A woody plant with usually a single stem growing to a height of at least two metres, or if multi-stemmed, then at least one vertical stem five centimetres in diameter at breast height”^73^. Note that this is an inclusive definition which includes monocots and tree ferns, as well as species that can occur both as tall single-stem and shrub-like multi-stem phenotypes.

To account for unequal representation of plots across biomes (Fig. 1), we used a reduced version of this database, down-sampled to a number of plots proportional to the land area covered by each of 14 biomes (Supplementary Table 1), while conserving as many tropical plots as possible. This ensured that we were not overrepresenting historically oversampled biomes, particularly in temperate regions. In addition, we preferentially retained invaded plots during this down-sampling to ensure adequate representation of invaded plots in the final dataset, with a maximum of half of the plots within a biome being invaded. This oversampling of invaded plots allowed for adequate representation of invaded and non-invaded plots in our analyses of non-native presence, and allowed sufficient data for our analyses of invasion severity, as these analyses only used data from plots that had non-native species invasions. Results were not qualitatively different if we did not preferentially retain invaded plots in our down-sampling (Extended Data Fig. 6 and Supplementary Table 8). Note also that the global mapping used the full dataset, with no subsampling. Prior to analyses, we also collapsed locations with multiple replicate plots and removed plots where phylogenetic of functional diversity could not be calculated for both native and full communities due to less than three species being present (see below).

### Non-native invasion metrics

We split our invasion metrics into the two broad categories of ‘non-native invasion’ (presence) and ‘invasion severity’. Specifically, using our data, we were able to determine for each plot (1) whether any non-native tree species were present (non-native presence); (2) the proportion of tree species that were non-native relative to total tree species (invasion severity, assessed via non-native richness)^23^; and (3) the proportion basal area of non-native tree species relative to total tree species basal area (invasion severity, assessed via non-native abundance). These metrics are congruent with recently proposed frameworks for measuring and reporting invasive plant species^74,75^. The metric of relative introduced species richness may be hypothesized to lead to a bias in detection of biotic resistance, with greater biotic resistance falsely detected in diverse communities, as these communities will have a lower proportion of non-native trees due to the higher denominator (total site diversity). However, use of the binomial approach in our GLM modelling of this proportion, as opposed to direct proportion, overcomes this limitation, as it uses raw counts of proportion, effectively weighting observations by the total species number in the community^23^.

### Climatic and anthropogenic variables

For climatic and anthropogenic variables, we relied on the Global Environmental Composite^76,77^. This global database contains spatially explicit geographic information system (GIS) layers of more than 260 unique environmental variables, encompassing climate, soil, land cover and land use, plant biomass, topography, human footprint, and disturbance^78,79^. Climate variables were extracted from the CHELSA (climatologies at high resolution for the earth’s land surface areas) dataset^78^, whereas soil variables were from the SoilGrids^80^ dataset. In addition, we created distance measures by calculating the spherical distance to shipping ports^81^ and airports^82^. All layers were standardized to a 30 arcsec resolution (~1 km^2^ at the equator), a resolution at which these variables have been shown to have an influence on plant biogeography and assembly patterns^83,84^. We chose model variables to represent both climate and soil properties that exhibited low collinearity for each of three datasets: global (all 14 biomes from Supplementary Table 1), temperate (temperate broadleaf, coniferous, grassland biomes) and tropical (tropical moist broadleaf, deciduous broadleaf, coniferous, and grassland biomes). We chose to use distinct variables rather than transforming them into principal component analysis axes for increased interpretability of these variables and their effects. Because variables exhibiting collinearity varied between the three datasets, the resulting models include different variable combinations. For all models, we used mean annual temperature (MAT), mean annual precipitation (MAP), distance to shipping ports^81^ (hereafter ‘ports’) and human population density^85^. For the global models, we used the following additional environmental variables: absolute depth to bedrock, coarse fragments, sand content and soil pH. For temperate models, we used absolute depth to bedrock, clay content, and soil pH as additional variables; for tropical models we used absolute depth to bedrock, soil organic content, and soil pH as additional variables. All soil variables used were determined at a depth of 0 cm, or the top layer of soil.

### Diversity metrics

**Extended Data Fig. 2:**
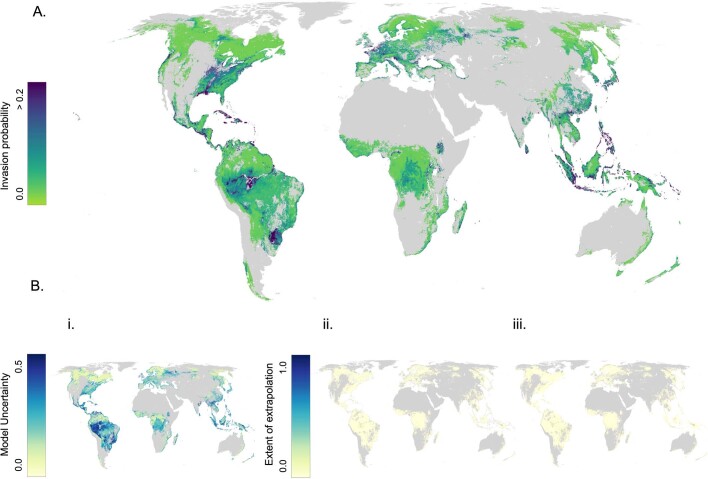
Map of non-native invasion probability inside the area of applicability. Map showing probability of non-native tree presence based on the probability output of the random forest classifier (A, total n = 368,030 plots, n per iteration = 10,000 plots) alongside maps showing uncertainty in predictions (B) including local uncertainty of invasion probability via bootstrapped coefficient of variation (i) and extent of extrapolation as percentage of bands outside univariate (ii) and multivariate (ii) training range. Regions outside the Area of Applicability are masked.

**Extended Data Fig. 3:**
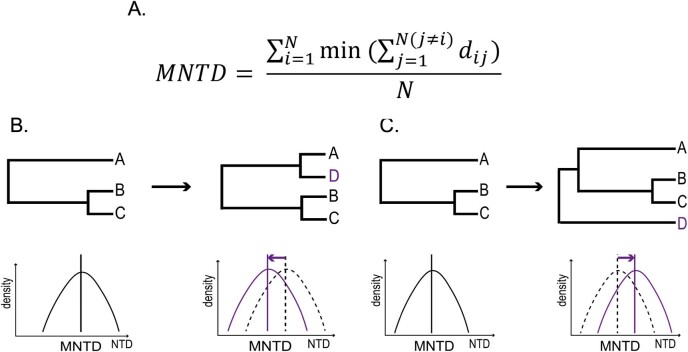
Mean nearest taxon distance (MNTD). Mean nearest taxon distance is the average distance to nearest neighbor by branch length on the tree, which represents redundancy in the community (A). For each species *i*, the sum of all shortest distances *d* to each other taxa *j* is calculated; these values are then averaged across the total species in the tree (N). If invasion occurs via non-natives being similar to the native community, this would lead to the expectation that MNTD decreases, increasing redundancy (B). Conversely, if non-native invasion occurs via non-natives being dissimilar to the native community, this would lead to the expectation that MNTD increases, reducing redundancy (C). Taxon D represents a non-native addition to the community.

**Extended Data Fig. 4:**
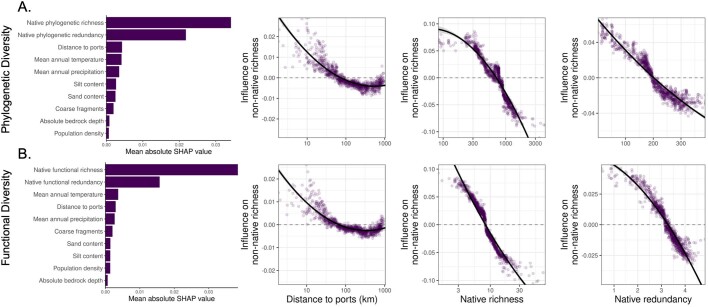
Native diversity mediates degree of non-native invasion. Variable importance (SHAP values) of all variables included in random forest models, ordered from greatest to least importance alongside influence of distance to ports, native richness and native redundancy on invasion severity (proportion of non-native plant species) for (A) phylogenetic diversity and (B) functional diversity global models (phylogenetic n = 3,498 plots; functional n = 3,368 plots). All results shown are from random forest models. Note that y-axis ranges differ among panels, with the variable importance plots representing the corresponding magnitude.

**Extended Data Fig. 5:**
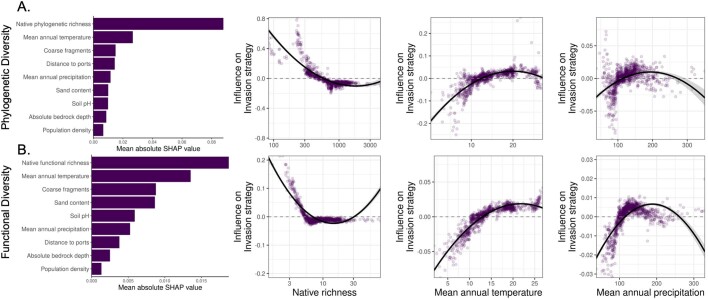
Variable importance for non-native invasion strategy. Variable importance (SHAP values) of all variables included in random forest models, ordered from greatest to least importance alongside influence of native richness, mean annual temperature and mean annual precipitation on invasion strategy for (A) phylogenetic diversity and (B) functional diversity global models (phylogenetic n = 3,498 plots; functional n = 3,368 plots). All results shown are from random forest models. Note that y-axis ranges differ among panels, with the variable importance plots representing the corresponding magnitude. Error bands represent 95% confidence intervals.

**Extended Data Fig. 6:**
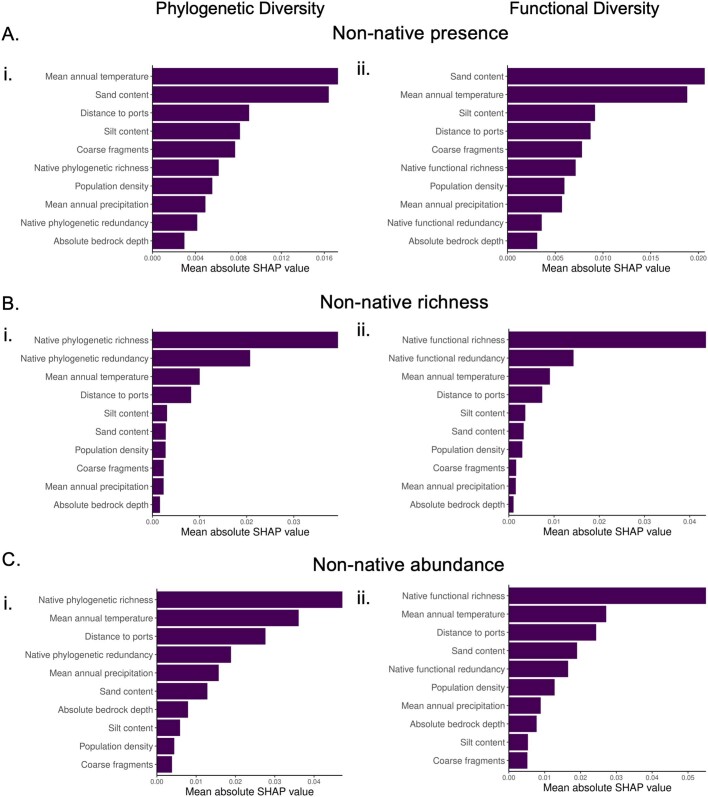
Variable importance for analyses using data down-sampled without preferentially retaining invaded plots. Variable importance (SHAP values) for all variables included in random forest models, ordered from greatest to least importance for (A) non-native presence, (B) richness, and (C) abundance, each for (i) phylogenetic diversity and (ii) functional diversity global models (presence: phylogenetic n = 18,898; functional n = 18,611, richness: phylogenetic n = 840 plots; functional n = 823 plots, abundance: phylogenetic n = 840 plots; functional n = 823 plots). All results shown are from random forest models with down-sampled data, but without preferentially retaining invaded plots. Error bands represent 95% confidence intervals.

We analysed data using either phylogenetic or functional diversity; these two approaches were chosen to be as analogous as possible. Phylogenetic alpha diversity explains the genetic relatedness of species within a community and is often assumed to represent a proxy for functional similarity across species within a community assemblage. Yet, congruency between these two metrics remains under debate^86,87^ and their role in invasion patterns remains untested; therefore, we focused on two major axes of diversity, explaining richness and divergence in the community across both phylogenetic and functional space^88^, capturing both evolutionary and ecological processes. For each native and entire tree community (native and non-native species), we calculated Faith’s phylogenetic diversity (phylogenetic richness) and mean nearest taxon distance (MNTD, phylogenetic redundancy; Extended Data Fig. 2). Entire tree community metrics were calculated on all species, whether they were matched to GloNAF and KEW or not; this included tree species which were identified to genus level. Faith’s phylogenetic diversity was calculated as the sum of the branch lengths on the phylogenetic tree of the species in the community; MNTD was calculated as the average distance to the nearest neighbour across the community. These metrics were calculated based on tree placement of taxa in a recently published reference backbone tree for plants^89^. Out of 13,345 starting taxa, a total of 12,325 were placed on the reference tree, with 4,960 placed at the species level and 7,365 placed at the genus level. We chose MNTD over other available metrics describing community divergence because we were interested in redundancy of the community, and this metric captures this best^24,90^. To enable a more intuitive understanding of this metric, we transformed each community-level value of MNTD to the maximum MNTD across all communities minus calculated MNTD. This transformed the maximum value to zero and all smaller values transformed to increasingly larger numbers, with higher MNTD values indicating a greater native redundancy, similar to the expected increased redundancy with greater phylogenetic richness (Faith’s phylogenetic diversity). To determine the non-native invasion strategy, or impact of non-natives on native MNTD, we calculated the difference between the native and non-native community relative to the native community alone. We used the following formula for non-native invasion strategy: (entire community MNTD – native community MNTD)/native community MNTD. When non-native invasion strategy was greater than zero, this indicated that the addition of the non-native species resulted in a more dissimilar community, whereas a non-native invasion strategy less than zero corresponded to the opposite.

For functional diversity, we calculated the analogous metrics using trait distance matrices instead of phylogenetic tree-based distances. We selected eight traits extracted from Maynard et al.^83^ that represented the major clusters of functional trait diversity, thereby capturing the full spectrum of tree form and function while minimizing correlation between traits. Maynard et al.^83^ used data from the TRY plant trait database to parametrize machine learning models to estimate the expression of 18 traits as a function of the local environment and/or phylogeny. The observed trait data underlying these models encompassed 491,001 unique observations across 13,189 species from 2,313 genera, with consistent representation across taxonomic orders. The resulting models were then used to generate trait estimates for 52,255 tree species, capturing approximately 80% of documented tree species^91^. Using this trait database, we were able to assign trait value to 81% of the tree species in GFBI reported to the species level. The eight traits we included in our metrics were chosen to include traits typically associated with plant invasion^28,92^ including those associated with dispersal, establishment, resource acquisition and competitive ability that represent the major trait clusters encompassing the full dimensionality of trait space from Maynard et al.^83^ The eight traits included in our study were the following: wood density, root depth, leaf nitrogen, leaf phosphorus, leaf area, tree height, seed dry mass, and bark thickness. All traits were log-transformed and normalized to allow for statistically valid comparisons^83^. To obtain functional diversity metrics analogous to those used for phylogenetic diversity, we used the dendrogram approach of Petchey and Gaston^93^. Specifically, for every plot we calculated the species-by-species trait distance matrix encompassing all eight traits, and then used hierarchical clustering to create a functional dendrogram. This dendrogram was subsequently used to calculate ‘functional richness’ (analogous to Faith’s phylogenetic diversity) and ‘functional redundancy’ (MNTD); we use this terminology for functional diversity to maintain naming of variables between phylogenetic and functional diversity analyses. Metrics were calculated in R using packages ape^94^, tidyverse^95^, abdiv^96^, doParallel^97^, foreach^98^ and pez^99^.

Because both functional and phylogenetic diversity metrics have unique limitations, we considered them both here so as to obtain a more robust view of underlying patterns and processes. The benefit of phylogenetic diversity is that it does not rely on imputed data, and thus it provides more consistent results with lower uncertainty. However, phylogenetic diversity is only a loose proxy for functioning, depending on the degree to which the functional traits of interest are phylogenetically conserved. Thus, as a complement of this, we also use imputed trait values to estimate functional diversity, which should better capture underlying functional differences across species, but which is subject to higher uncertainty relative to phylogeny (or measured trait values), and may omit rare and potentially functionally unique species. Thus, by simultaneously considering both functional and phylogenetic diversity and showing that these metrics yield consistent global trends, our approach provides consistent evidence that these patterns are robust to the limitations of either approach taken individually.

### Statistical analyses

We combined random forest^100^ and GLM approaches to answer our focal questions. Specifically, we used random forest models to visualize patterns and determine variable importance, while GLMs were used to assess statistical significance and directionality of patterns. We first tested for environmental and anthropogenic drivers of non-native invasion, including non-native presence and invasion severity (non-native richness, non-native abundance). Our independent variables included either phylogenetic or functional metrics, climate and soil variables, and human impact variables. Next, we tested the impact of these variables on non-native invasion strategy (difference in MNTD due to non-natives). We focused on addressing specific hypotheses related to drivers of non-native invasion and invasion strategy. We acknowledge the importance of other variables, and therefore included them in our models, but do not interpret each variable.

Random forest models and GLMs used the same model designs. Models predicting non-native presence as well as invasion severity, for both non-native richness and abundance, included independent predictor variables of native diversity and native redundancy, as well as climate and human driver variables detailed in *‘Climatic and anthropogenic variables’*. For comparison, we repeated these models with native tree species richness in place of both diversity variables (richness and redundancy), as species richness is commonly used in the invasion literature when testing for biotic resistance^23,34,35^. Finally, we used an adapted version of the random forest models, removing diversity variables, to assess probability of locations with non-native trees globally and generate an associated map (Extended Data Fig. 1).

To account for spatial autocorrelation in the modelling step, we used residual autocovariates (RACs)^101,102^. First, we used simple linear regression to determine the range of spatial autocorrelation for the models with continuous outcomes (invasion severity and invasion strategy). We then assessed residual spatial autocorrelation using correlelogram plots using the ncf^103^ package in R, which showed that residual correlation was consistently negligible beyond 250 km, which was also applied to the models with binary outcomes (non-native presence). Using this buffer distance, we generated RAC values using the autocov_dist() function in the spdep package^70,104^, which determines an inverse distance weighted residual value for each data point in the 250 km neighbourhood. RAC incorporates the spatial signature of the model residuals, relative to the model without any spatial autocorrelation correction, into a variable that is included in each model^101,102^. The result is an inverse distance weighted residual value for each data point in the 250 km neighbourhood, which we used as continuous predictors in both the linear and random forest models.

Random forest models were used primarily to assess variable importance and influence. Specifically, we used Shapley additive explanations (SHAP) values to infer variable importance in the model outcome^105,106^. SHAP values are a machine learning analogue of partial regression, quantifying the relative importance of each variable on the outcome, accounting for all other variables in the model. To estimate the SHAP values, random forest models were fit in R using the ranger package^107^, using default hyperparameters (500 trees, observations sampled with replacement, number of variables per split equal to the square root of the number of predictors, a minimum of 5 observations per node). We then used the fastshap package^108^ to estimate approximate SHAP values for each predictor, using *n* = 100 simulations. The overall variable importance was taken as the sum of the absolute value of the SHAP values, and the marginal effect of each variable was visualized by plotting the covariate versus the corresponding SHAP value for each observation.

To account for spatial autocorrelation in the accuracy assessment of random forest models, we implemented spatially-buffered leave-one-out cross-validation (LOO-CV) to obtain conservative lower-bound accuracy measures^109^. To do this, we first randomly selected a focal observation as the test data, and then we omitted all observations within a 250 km buffer distance around this observation. The remaining data were used to train the model, and the resulting fit was used to predict outcome for the withheld focal observation. This was repeated 500 times for each model, each time selecting a new focal point and predicting its outcome using the 250 km spatially-buffered training set. The resulting accuracy measures were calculated on the set of 500 out-of-fit predictions. For continuous variables, we estimated accuracy using the cross-validated coefficient of determination relative to the one-to-one line (termed VEcv^110^), denoted simply *R*^2^ here, and for binary outcomes we used area under the ROC curve (AUC), which quantifies the ability of the classifier to distinguish between classes, and serves as an assessment of model performance.

To create a global map of invasion probability and its local uncertainty, we used a repeated prediction approach in Google Earth Engine^60^ (Extended Data Fig. 1a; AUC of spatial cross-validation = 0.84 ± 0.04, mean F1 score of non-native presence = 0.36). This repeated prediction approach used the full dataset without any down-sampling. To our knowledge, no global maps on phylogenetic or functional diversity metrics exist, so we were unable to include these diversity metrics in the random forest model for mapping; therefore, these models include the same covariates as the other models except diversity metrics. We thought it reasonable to exclude diversity metrics in this analysis as distance to ports is the most important driver of invasion probability, while native diversity is less important. After aggregating samples within the 30-arcsec pixels, 368,030 data points remained for our repeated prediction approach. We first trained 50 random forest models on stratified bootstrapped samples with a total of 10,000 data points each, using biome as stratification category; this allowed us to repeatedly predict the probability of non-native presence for each terrestrial pixel on Earth. The resulting 50 predictions were used to create per-pixel mean and coefficient of variation maps of the probability of non-native presence, with probabilities calibrated using Platt scaling^111,112^. These two maps allow us to investigate the patterns of invasion and the regions of uncertainty in the predictions. Next, the extrapolation extent was estimated as a per-pixel percentage of predictor variables, and interactions of predictor variables, outside of the training range, in univariate and multivariate space, respectively (Extended Data Fig. 1b)^60^. In addition, to account for gaps in predictor space, we estimated the Area of Applicability^113^, used to mark regions of extrapolation in this map. All maps are restricted to regions with a minimum of 10% forest cover^114^.

GLM models were used to estimate statistical parameters and conduct statistical tests. All GLM models included the same variables as those in the random forest models. In the models predicting non-native presence, we used a binomial distribution and logit link. For non-native abundance, we used a beta regression approach to predict the proportion of non-native basal area, as a method of modelling proportions between 0 and 1. We could not use a binomial GLM analogous to that used for non-native abundance because basal area measurements were not whole numbers and we wanted to retain all information in the data. Finally, to account for spatial autocorrelation and non-independently distributed residuals, we employed the inclusion of RACs as described above. These models were repeated separately for temperate and tropical bioclimatic zones, but results were qualitatively similar to the global model, so we report only global results here. All GLM results can be found in Supplementary Tables 3–5. GLMs were run in R (v. 4.2.2)^115^ using lme4^116^, lmerTest^117^, and betareg^118^, while visualizations for these models used ggplot2^119^; tidyverse^95^ was used throughout as well.

Because invasion of non-native species may alter the native diversity of the site into which they invade, we conducted a sensitivity test using plots where we had data across two time points to incorporate this effect. We first took all plots for which we had two time points, where the first time point represented a fully native community (that is, no presence of non-natives; *n* = 8,221 plots). We then modelled the per cent change of species richness in each plot from this uninvaded first time point to a later time point. Our predictor variables included final invasion status (non-natives present or not) to determine the impact of invasion on per cent change of species richness, along with all climate, soil, and anthropogenic impact variables we included in other global models. We extracted the coefficient of final invasion status (along with upper and lower confidence ranges), which quantifies the per cent change in richness due to invasion, and we used this to update the native species richness of the full global dataset. We then used these coefficients to estimate the pre-invasion native diversity for each plot in the global dataset by adding the corresponding species change resulting from invasion. Finally, we reran our global analysis with this updated pre-invasion native diversity. The relative contribution of native species loss to biotic resistance was calculated by comparing the relative change in the richness coefficient for each of the updated models relative to the original model (Supplementary Table 9).

Non-native invasion strategy was predicted using the difference in redundancy (MNTD) in the tree community due to invasion. We included the same variables as in the previous set of models, except native redundancy, as this is integrated in our response variable and therefore would exhibit high collinearity. In GLM models, we tested for the interaction between MAP and MAT to detect potential non-additive environmental filtering effects of these two dominant climate variables. In addition, we tested for the interaction between each MAP and MAT with distance to ports, to examine whether this important anthropogenic driver modified main ecological relationships. Final reported models are those resulting from a process of first creating a full model with all interactions, and subsequently removing nonsignificant interactions. All GLM results for invasion strategy can be found in Supplementary Table 7.

### Reporting summary

Further information on research design is available in the Nature Portfolio Reporting Summary linked to this article.

## Online content

Any methods, additional references, Nature Portfolio reporting summaries, source data, extended data, supplementary information, acknowledgements, peer review information; details of author contributions and competing interests; and statements of data and code availability are available at 10.1038/s41586-023-06440-7.

### Supplementary information

Supplementary Tables This file contains Supplementary Tables 1–9.

Reporting Summary

## Data Availability

Data used in this study can be found in cited references for the Global Naturalized Alien Flora (GloNAF) database^6^ (non-native status), the KEW Plants of the World database^5^ (native ranges) and the Global Environmental Composite^63,77^ (environmental data layers). Plant trait data were extracted from Maynard et al.^78^. Data from the Global Forest Biodiversity Initiative (GFBI) database^57^ are not available due to data privacy and sharing restrictions, but can be obtained upon request via Science-I (https://science-i.org/) or GFBI (gfbinitiative.org) and an approval from data contributors.

## Code availability

All code used to complete analyses for the manuscript is available at the following link: https://github.com/thomaslauber/Global-Tree-Invasion. Data analyses were conducted and were visualizations generated in R (v. 4.2.2), Python (v. 3.9.7), Google Earth Engine (earthengine-api 0.1.306), QGIS-LTR (v. 3.16.7) and the ETH Zurich Euler cluster.
