# Author Correction: Native diversity buffers against severity of non-native tree invasions

**DOI:** 10.1038/s41586-023-06654-9

**Published:** 2023-09-26

**Authors:** Camille S. Delavaux, Thomas W. Crowther, Constantin M. Zohner, Niamh M. Robmann, Thomas Lauber, Johan van den Hoogen, Sara Kuebbing, Jingjing Liang, Sergio de-Miguel, Gert-Jan Nabuurs, Peter B. Reich, Meinrad Abegg, Yves C. Adou Yao, Giorgio Alberti, Angelica M. Almeyda Zambrano, Braulio Vilchez Alvarado, Esteban Alvarez-Dávila, Patricia Alvarez-Loayza, Luciana F. Alves, Christian Ammer, Clara Antón-Fernández, Alejandro Araujo-Murakami, Luzmila Arroyo, Valerio Avitabile, Gerardo A. Aymard, Timothy R. Baker, Radomir Bałazy, Olaf Banki, Jorcely G. Barroso, Meredith L. Bastian, Jean-Francois Bastin, Luca Birigazzi, Philippe Birnbaum, Robert Bitariho, Pascal Boeckx, Frans Bongers, Olivier Bouriaud, Pedro H. S. Brancalion, Susanne Brandl, Roel Brienen, Eben N. Broadbent, Helge Bruelheide, Filippo Bussotti, Roberto Cazzolla Gatti, Ricardo G. César, Goran Cesljar, Robin Chazdon, Han Y. H. Chen, Chelsea Chisholm, Hyunkook Cho, Emil Cienciala, Connie Clark, David Clark, Gabriel D. Colletta, David A. Coomes, Fernando Cornejo Valverde, José J. Corral-Rivas, Philip M. Crim, Jonathan R. Cumming, Selvadurai Dayanandan, André L. de Gasper, Mathieu Decuyper, Géraldine Derroire, Ben DeVries, Ilija Djordjevic, Jiri Dolezal, Aurélie Dourdain, Nestor Laurier Engone Obiang, Brian J. Enquist, Teresa J. Eyre, Adandé Belarmain Fandohan, Tom M. Fayle, Ted R. Feldpausch, Leandro V. Ferreira, Markus Fischer, Christine Fletcher, Lorenzo Frizzera, Javier G. P. Gamarra, Damiano Gianelle, Henry B. Glick, David J. Harris, Andrew Hector, Andreas Hemp, Geerten Hengeveld, Bruno Hérault, John L. Herbohn, Martin Herold, Annika Hillers, Eurídice N. Honorio Coronado, Cang Hui, Thomas T. Ibanez, Iêda Amaral, Nobuo Imai, Andrzej M. Jagodziński, Bogdan Jaroszewicz, Vivian Kvist Johannsen, Carlos A. Joly, Tommaso Jucker, Ilbin Jung, Viktor Karminov, Kuswata Kartawinata, Elizabeth Kearsley, David Kenfack, Deborah K. Kennard, Sebastian Kepfer-Rojas, Gunnar Keppel, Mohammed Latif Khan, Timothy J. Killeen, Hyun Seok Kim, Kanehiro Kitayama, Michael Köhl, Henn Korjus, Florian Kraxner, Diana Laarmann, Mait Lang, Simon L. Lewis, Huicui Lu, Natalia V. Lukina, Brian S. Maitner, Yadvinder Malhi, Eric Marcon, Beatriz Schwantes Marimon, Ben Hur Marimon-Junior, Andrew R. Marshall, Emanuel H. Martin, Olga Martynenko, Jorge A. Meave, Omar Melo-Cruz, Casimiro Mendoza, Cory Merow, Abel Monteagudo Mendoza, Vanessa S. Moreno, Sharif A. Mukul, Philip Mundhenk, María Guadalupe Nava-Miranda, David Neill, Victor J. Neldner, Radovan V. Nevenic, Michael R. Ngugi, Pascal A. Niklaus, Jacek Oleksyn, Petr Ontikov, Edgar Ortiz-Malavasi, Yude Pan, Alain Paquette, Alexander Parada-Gutierrez, Elena I. Parfenova, Minjee Park, Marc Parren, Narayanaswamy Parthasarathy, Pablo L. Peri, Sebastian Pfautsch, Oliver L. Phillips, Nicolas Picard, Maria Teresa T. F. Piedade, Daniel Piotto, Nigel C. A. Pitman, Irina Polo, Lourens Poorter, Axel D. Poulsen, Hans Pretzsch, Freddy Ramirez Arevalo, Zorayda Restrepo-Correa, Mirco Rodeghiero, Samir G. Rolim, Anand Roopsind, Francesco Rovero, Ervan Rutishauser, Purabi Saikia, Christian Salas-Eljatib, Philippe Saner, Peter Schall, Dmitry Schepaschenko, Michael Scherer-Lorenzen, Bernhard Schmid, Jochen Schöngart, Eric B. Searle, Vladimír Seben, Josep M. Serra-Diaz, Douglas Sheil, Anatoly Z. Shvidenko, Javier E. Silva-Espejo, Marcos Silveira, James Singh, Plinio Sist, Ferry Slik, Bonaventure Sonké, Alexandre F. Souza, Stanislaw Miscicki, Krzysztof J. Stereńczak, Jens-Christian Svenning, Miroslav Svoboda, Ben Swanepoel, Natalia Targhetta, Nadja Tchebakova, Hans ter Steege, Raquel Thomas, Elena Tikhonova, Peter M. Umunay, Vladimir A. Usoltsev, Renato Valencia, Fernando Valladares, Fons van der Plas, Tran Van Do, Michael E. van Nuland, Rodolfo M. Vasquez, Hans Verbeeck, Helder Viana, Alexander C. Vibrans, Simone Vieira, Klaus von Gadow, Hua-Feng Wang, James V. Watson, Gijsbert D. A. Werner, Susan K. Wiser, Florian Wittmann, Hannsjoerg Woell, Verginia Wortel, Roderik Zagt, Tomasz Zawiła-Niedźwiecki, Chunyu Zhang, Xiuhai Zhao, Mo Zhou, Zhi-Xin Zhu, Irie C. Zo-Bi, Daniel S. Maynard

**Affiliations:** 1 Institute of Integrative Biology, ETH Zurich (Swiss Federal Institute of Technology), Zurich, Switzerland; 2 The Forest School at The Yale School of the Environment, Yale University, New Haven, CT USA; 3 Department of Forestry and Natural Resources, Purdue University, West Lafayette, IN USA; 4 Department of Crop and Forest Sciences, University of Lleida, Lleida, Spain; 5 Joint Research Unit CTFC–AGROTECNIO–CERCA, Solsona, Spain; 6 Wageningen University and Research, Wageningen, The Netherlands; 7 Department of Forest Resources, University of Minnesota, St Paul, MN USA; 8 Hawkesbury Institute for the Environment, Western Sydney University, Penrith, New South Wales Australia; 9 Institute for Global Change Biology, and School for Environment and Sustainability, University of Michigan, Ann Arbor, MI USA; 10 Swiss Federal Institute for Forest, Snow and Landscape Research WSL, Birmensdorf, Switzerland; 11 UFR Biosciences, University Félix Houphouët-Boigny, Abidjan, Côte d’Ivoire; 12 Department of Agricultural, Food, Environmental and Animal Sciences, University of Udine, Udine, Italy; 13 Faculty of Science and Technology, Free University of Bolzano, Bolzano, Italy; 14 Spatial Ecology and Conservation Laboratory, Department of Tourism, Recreation and Sport Management, University of Florida, Gainesville, FL USA; 15 Forestry School, Tecnológico de Costa Rica TEC, Cartago, Costa Rica; 16 Fundacion ConVida, Universidad Nacional Abierta y a Distancia, UNAD, Medellin, Colombia; 17 Field Museum of Natural Histiory, Chicago, IL USA; 18 Center for Tropical Research, Institute of the Environment and Sustainability, UCLA, Los Angeles, CA USA; 19 Silviculture and Forest Ecology of the Temperate Zones, University of Göttingen, Göttingen, Germany; 20 Division of Forest and Forest Resources, Norwegian Institute of Bioeconomy Research (NIBIO), Ås, Norway; 21 Museo de Historia Natural Noel kempff Mercado, Santa Cruz, Bolivia; 22 European Commission, Joint Research Center, Ispra, Italy; 23 UNELLEZ-Guanare, Programa de Ciencias del Agro y el Mar, Herbario Universitario (PORT), Portuguesa, Venezuela; 24 Compensation International S. A. Ci Progress–GreenLife, Bogotá, Colombia; 25 School of Geography, University of Leeds, Leeds, UK; 26 Department of Geomatics, Forest Research Institute, Raszyn, Poland; 27 Naturalis Biodiversity Center, Leiden, The Netherlands; 28 Centro Multidisciplinar, Universidade Federal do Acre, Rio Branco, Brazil; 29 Proceedings of the National Academy of Sciences, Washington, DC USA; 30 Department of Evolutionary Anthropology, Duke University, Durham, NC USA; 31 TERRA Teach and Research Centre, Gembloux Agro Bio-Tech, University of Liege, Liege, Belgium; 32 United Nation Framework Convention on Climate Change, Bonn, Germany; 33 Institut Agronomique néo-Calédonien (IAC), Nouméa, New Caledonia; 34 AMAP, University of Montpellier, Montpellier, France; 35 AMAP, University of Montpellier, CIRAD, CNRS, INRAE, IRD, Montpellier, France; 36 Institute of Tropical Forest Conservation, Mbarara University of Sciences and Technology, Mbarara, Uganda; 37 Isotope Bioscience Laboratory–ISOFYS, Ghent University, Ghent, Belgium; 38 Integrated Center for Research, Development and Innovation in Advanced Materials, Nanotechnologies, and Distributed Systems for Fabrication and Control (MANSiD), Stefan cel Mare University of Suceava, Suceava, Romania; 39 Department of Forest Sciences, Luiz de Queiroz College of Agriculture, University of São Paulo, Piracicaba, Brazil; 40 Bavarian State Institute of Forestry, Freising, Germany; 41 Spatial Ecology and Conservation Laboratory, School of Forest Resources and Conservation, University of Florida, Gainesville, FL USA; 42 Institute of Biology, Geobotany and Botanical Garden, Martin Luther University Halle-Wittenberg, Halle-Wittenberg, Germany; 43 German Centre for Integrative Biodiversity Research (iDiv) Halle-Jena-Leipzig, Leipzig, Germany; 44 Department of Agriculture, Food, Environment and Forest (DAGRI), University of Firenze, Florence, Italy; 45 Department of Biological, Geological, and Environmental Sciences, University of Bologna, Bologna, Italy; 46 Department of Spatial Regulation, GIS and Forest Policy, Institute of Forestry, Belgrade, Serbia; 47 Department of Ecology and Evolutionary Biology, University of Connecticut, Storrs, CT USA; 48 Forest Research Institute, University of the Sunshine Coast, Sippy Downs, Queensland Australia; 49 Faculty of Natural Resources Management, Lakehead University, Thunder Bay, Ontario Canada; 50 Division of Forest Resources Information, Korea Forest Promotion Institute, Seoul, South Korea; 51 IFER–Institute of Forest Ecosystem Research, Jilove u Prahy, Czech Republic; 52 Global Change Research Institute CAS, Brno, Czech Republic; 53 Nicholas School of the Environment, Duke University, Durham, NC USA; 54 Department of Biology, University of Missouri-St Louis, St Louis, MO USA; 55 Programa de Pós-graduação em Biologia Vegetal, Instituto de Biologia, Universidade Estadual de Campinas, Campinas, Brazil; 56 Department of Plant Sciences and Conservation Research Institute, University of Cambridge, Cambridge, UK; 57 Andes to Amazon Biodiversity Program, Madre de Dios, Peru; 58 Facultad de Ciencias Forestales y Ambientales, Universidad Juárez del Estado de Durango, Durango, Mexico; 59 Department of Biology, West Virginia University, Morgantown, WV USA; 60 Department of Physical and Biological Sciences, The College of Saint Rose, Albany, NY USA; 61 Biology Department, Centre for Structural and Functional Genomics, Concordia University, Montreal, Quebec Canada; 62 Natural Science Department, Universidade Regional de Blumenau, Blumenau, Brazil; 63 World Agroforestry (ICRAF), Nairobi, Kenya; 64 Cirad, UMR EcoFoG (AgroParisTech, CNRS, INRAE), Université des Antilles, Université de la Guyane, Campus Agronomique, Kourou, France; 65 Department of Geographical Sciences, University of Maryland, College Park, MD USA; 66 Institute of Forestry, Belgrade, Serbia; 67 Institute of Botany, The Czech Academy of Sciences, Třeboň, Czech Republic; 68 Department of Botany, Faculty of Science, University of South Bohemia, České Budějovice, Czech Republic; 69 IRET, Herbier National du Gabon (CENAREST), Libreville, Gabon; 70 Department of Ecology and Evolutionary Biology, University of Arizona, Tucson, AZ USA; 71 The Santa Fe Institute, Santa Fe, NM USA; 72 Queensland Herbarium, Department of Environment and Science, Toowong, Queensland Australia; 73 Ecole de Foresterie et Ingénierie du Bois, Université Nationale d’Agriculture, Kétou, Benin; 74 School of Biological and Behavioural Sciences, Queen Mary University of London, London, UK; 75 Biology Centre of the Czech Academy of Sciences, Institute of Entomology, Ceske Budejovice, Czech Republic; 76 Geography, College of Life and Environmental Sciences, University of Exeter, Exeter, UK; 77 Museu Paraense Emílio Goeldi. Coordenação de Ciências da Terra e Ecologia, Belém, Pará Brazil; 78 Institute of Plant Sciences, University of Bern, Bern, Switzerland; 79 Forest Research Institute Malaysia, Kuala Lumpur, Malaysia; 80 Research and Innovation Center, Fondazione Edmund Mach, San Michele All’adige, Italy; 81 Forestry Division, Food and Agriculture Organization of the United Nations, Rome, Italy; 82 Glick Designs LLC, Hadley, MA USA; 83 Royal Botanic Garden Edinburgh, Edinburgh, UK; 84 Department of Plant Sciences, University of Oxford, Oxford, UK; 85 Department of Plant Systematics, University of Bayreuth, Bayreuth, Germany; 86 Cirad, UPR Forêts et Sociétés, University of Montpellier, Montpellier, France; 87 Department of Forestry and Environment, National Polytechnic Institute (INP-HB), Yamoussoukro, Côte d’Ivoire; 88 Tropical Forests and People Research Centre, University of the Sunshine Coast, Maroochydore, Queensland Australia; 89 Centre for Conservation Science, The Royal Society for the Protection of Birds, Sandy, UK; 90 Wild Chimpanzee Foundation, Liberia Office, Monrovia, Liberia; 91 Instituto de Investigaciones de la Amazonía Peruana, Iquitos, Peru; 92 Centre for Invasion Biology, Department of Mathematical Sciences, Stellenbosch University, Stellenbosch, South Africa; 93 Theoretical Ecology Unit, African Institute for Mathematical Sciences, Cape Town, South Africa; 94 National Institute of Amazonian Research, Manaus, Brazil; 95 Department of Forest Science, Tokyo University of Agriculture, Tokyo, Japan; 96 Institute of Dendrology, Polish Academy of Sciences, Kórnik, Poland; 97 Poznań University of Life Sciences, Department of Game Management and Forest Protection, Poznań, Poland; 98 Faculty of Biology, Białowieża Geobotanical Station, University of Warsaw, Białowieża, Poland; 99 Department of Geosciences and Natural Resource Management, University of Copenhagen, Copenhagen, Denmark; 100 Department of Plant Biology, Institute of Biology, University of Campinas, UNICAMP, Campinas, Brazil; 101 School of Biological Sciences, University of Bristol, Bristol, UK; 102 Forestry Faculty, Bauman Moscow State Technical University, Mytischi, Russia; 103 Field Museum of Natural History, Chicago, IL USA; 104 CAVElab-Computational and Applied Vegetation Ecology, Department of Environment, Ghent University, Ghent, Belgium; 105 CTFS-ForestGEO, Smithsonian Tropical Research Institute, Balboa, Panama; 106 Department of Physical and Environmental Sciences, Colorado Mesa University, Grand Junction, CO USA; 107 UniSA STEM and Future Industries Institute, University of South Australia, Adelaide, South Australia Australia; 108 Department of Botany, Dr Harisingh Gour Vishwavidyalaya (A Central University), Sagar, India; 109 Department of Agriculture, Forestry and Bioresources, Seoul National University, Seoul, South Korea; 110 Interdisciplinary Program in Agricultural and Forest Meteorology, Seoul National University, Seoul, South Korea; 111 National Center for Agro Meteorology, Seoul, South Korea; 112 Research Institute for Agriculture and Life Sciences, Seoul National University, Seoul, South Korea; 113 Graduate School of Agriculture, Kyoto University, Kyoto, Japan; 114 Institute for World Forestry, University of Hamburg, Hamburg, Germany; 115 Institute of Forestry and Engineering, Estonian University of Life Sciences, Tartu, Estonia; 116 Biodiversity and Natural Resources Program, International Institute for Applied Systems Analysis (IIASA), Laxenburg, Austria; 117 Department of Geography, University College London, London, UK; 118 Faculty of Forestry, Qingdao Agricultural University, Qingdao, China; 119 Center for Forest Ecology and Productivity, Russian Academy of Sciences, Moscow, Russia; 120 Environmental Change Institute, School of Geography and the Environment, University of Oxford, Oxford, UK; 121 AgroParisTech, UMR-AMAP, Cirad, CNRS, INRA, IRD, Université de Montpellier, Montpellier, France; 122 Departamento de Ciências Biológicas, Universidade do Estado de Mato Grosso, Nova Xavantina, Brazil; 123 Department of Environment and Geography, University of York, York, UK; 124 Flamingo Land, Malton, UK; 125 Department of Wildlife Management, College of African Wildlife Management, Mweka, Tanzania; 126 Departamento de Ecología y Recursos Naturales, Facultad de Ciencias, Universidad Nacional Autónoma de México, Mexico City, Mexico; 127 Universidad del Tolima, Ibagué, Colombia; 128 Colegio de Profesionales Forestales de Cochabamba, Cochabamba, Bolivia; 129 Jardín Botánico de Missouri, Pasco, Peru; 130 Universidad Nacional de San Antonio Abad del Cusco, Cusco, Peru; 131 Department of Environment and Development Studies, United International University, Dhaka, Bangladesh; 132 Laboratorio de geomática, Instituto de Silvicultura e Industria de la Madera, Universidad Juárez del Estado de Durango, Durango, Mexico; 133 Programa de doctorado en Ingeniería para el desarrollo rural y civil, Escuela de Doctorado Internacional de la Universidad de Santiago de Compostela, Santiago de Compostela, Spain; 134 Department of Environment and Development Studies, United International University, Dhaka, Bangladesh; 135 Universidad Estatal Amazónica, Puyo, Pastaza Ecuador; 136 Department of Evolutionary Biology and Environmental Studies, University of Zürich, Zurich, Switzerland; 137 Climate, Fire, and Carbon Cycle Sciences, USDA Forest Service, Durham, NC USA; 138 Centre for Forest Research, Université du Québec à Montréal, Montreal, Quebec Canada; 139 V. N. Sukachev Institute of Forest, FRC KSC, Siberian Branch of the Russian Academy of Sciences, Krasnoyarsk, Russia; 140 Forest Ecology and Forest Management Group, Wageningen University and Research, Wageningen, The Netherlands; 141 Department of Ecology and Environmental Sciences, Pondicherry University, Puducherry, India; 142 Instituto Nacional de Tecnología Agropecuaria (INTA), Universidad Nacional de la Patagonia Austral (UNPA), Consejo Nacional de Investigaciones Científicas y Tecnicas (CONICET), Río Gallegos, Argentina; 143 School of Social Sciences (Urban Studies), Western Sydney University, Penrith, New South Wales Australia; 144 Forestry Department, Food and Agriculture Organization of the United Nations, Rome, Italy; 145 Instituto Nacional de Pesquisas da Amazônia, Manaus, Brazil; 146 Laboratório de Dendrologia e Silvicultura Tropical, Centro de Formação em Ciências Agroflorestais, Universidade Federal do Sul da Bahia, Itabuna, Brazil; 147 Jardín Botánico de Medellín, Medellin, Colombia; 148 Chair for Forest Growth and Yield Science, TUM School for Life Sciences, Technical University of Munich, Munich, Germany; 149 Universidad Nacional de la Amazonía Peruana, Iquitos, Peru; 150 Servicios Ecosistémicos y Cambio Climático (SECC), Fundación Con Vida & Corporación COL-TREE, Medellín, Colombia; 151 Centro Agricoltura, Alimenti, Ambiente, University of Trento, San Michele All’adige, Italy; 152 Department of Biological Sciences, Boise State University, Boise, ID USA; 153 Department of Biology, University of Florence, Florence, Italy; 154 Tropical Biodiversity, MUSE–Museo delle Scienze, Trento, Italy; 155 Info Flora, Geneva, Switzerland; 156 Department of Environmental Sciences, Central University of Jharkhand, Ranchi, Jharkhand India; 157 Centro de Modelación y Monitoreo de Ecosistemas, Universidad Mayor, Santiago, Chile; 158 Vicerrectoria de Investigacion y Postgrado, Universidad de La Frontera, Temuco, Chile; 159 Depto. de Silvicultura y Conservacion de la Naturaleza, Universidad de Chile, Temuco, Chile; 160 Datascientist.ch, Wallisellen, Switzerland; 161 Siberian Federal University, Krasnoyarsk Russian Federation, Krasnoyarsk, Russia; 162 Geobotany, Faculty of Biology, University of Freiburg, Freiburg im Breisgau, Germany; 163 National Forest Centre, Forest Research Institute Zvolen, Zvolen, Slovakia; 164 Université de Lorraine, AgroParisTech, INRAE, Silva, Nancy, France; 165 Center for Ecological Dynamics in a Novel Biosphere (ECONOVO) and Center for Biodiversity Dynamics in a Changing World (BIOCHANGE), Department of Biology, Aarhus University, Aarhus, Denmark; 166 Forest Ecology and Forest Management, Wageningen University and Research, Wageningen, The Netherlands; 167 Faculty of Environmental Sciences and Natural Resource Management, Norwegian University of Life Sciences, Ås, Norway; 168 Departamento de Biología, Universidad de la Serena, La Serena, Chile; 169 Centro de Ciências Biológicas e da Natureza, Universidade Federal do Acre, Rio Branco, Acre Brazil; 170 Guyana Forestry Commission, Georgetown, France; 171 Environmental and Life Sciences, Faculty of Science, Universiti Brunei Darussalam, Bandar Seri Begawan, Brunei; 172 Plant Systematic and Ecology Laboratory, Department of Biology, Higher Teachers’ Training College, University of Yaoundé I, Yaoundé, Cameroon; 173 Departamento de Ecologia, Universidade Federal do Rio Grande do Norte, Natal, Rio Grande do Norte Brazil; 174 Warsaw University of Life Sciences, Warsaw, Poland; 175 Section for Ecoinformatics & Biodiversity, Department of Biology, Aarhus University, Aarhus, Denmark; 176 Faculty of Forestry and Wood Sciences, Czech University of Life Sciences, Prague, Czech Republic; 177 Wildlife Conservation Society, Vientiane, Laos; 178 Quantitative Biodiversity Dynamics, Betafaculty, Utrecht University, Utrecht, The Netherlands; 179 Iwokrama International Centre for Rainforest Conservation and Development (IIC), Georgetown, Guyana; 180 School of Forestry and Environmental Studies, Yale University, New Haven, CT USA; 181 Botanical Garden of Ural Branch of Russian Academy of Sciences, Ural State Forest Engineering University, Yekaterinburg, Russia; 182 Pontificia Universidad Católica del Ecuador, Quito, Ecuador; 183 LINCGlobal, Museo Nacional de Ciencias Naturales, CSIC, Madrid, Spain; 184 Plant Ecology and Nature Conservation Group, Wageningen University, Wageningen, The Netherlands; 185 Silviculture Research Institute, Vietnamese Academy of Forest Sciences, Hanoi, Vietnam; 186 Department of Biology, Stanford University, Stanford, CA USA; 187 Centre for the Research and Technology of Agro-Environmental and Biological Sciences, CITAB, University of Trás-os-Montes and Alto Douro, UTAD, Viseu, Portugal; 188 Department of Ecology and Sustainable Agriculture, Agricultural High School, Polytechnic Institute of Viseu, Viseu, Portugal; 189 Department of Forest Engineering Universidade Regional de Blumenau, Blumenau, Brazil; 190 Environmental Studies and Research Center, University of Campinas, UNICAMP, Campinas, Brazil; 191 Department of Forest and Wood Science, University of Stellenbosch, Stellenbosch, South Africa; 192 Key Laboratory of Tropical Biological Resources, Ministry of Education, School of Life and Pharmaceutical Sciences, Hainan University, Haikou, China; 193 Division of Forestry and Natural Resources, West Virginia University, Morgantown, WV USA; 194 Department of Zoology, University of Oxford, Oxford, UK; 195 Manaaki Whenua–Landcare Research, Lincoln, New Zealand; 196 Department of Wetland Ecology, Institute for Geography and Geoecology, Karlsruhe Institute for Technology, Karlsruhe, Germany; 197 Independent Researcher, Bad Aussee, Austria; 198 Centre for Agricultural Research in Suriname (CELOS), Paramaribo, Suriname; 199 Tropenbos International, Wageningen, The Netherlands; 200 Polish State Forests, Coordination Center for Environmental Projects, Warsaw, Poland; 201 Research Center of Forest Management Engineering of State Forestry and Grassland Administration, Beijing Forestry University, Beijing, China; 202 Department of Genetics, Evolution, and Environment, University College London, London, UK

**Keywords:** Invasive species, Biogeography, Forest ecology

Correction to: *Nature* 10.1038/s41586-023-06440-7 Published online 23 August 2023

In the version of the article initially published, Stanislaw Miscicki’s name incorrectly appeared as Miscicki Stanislaw. Additionally, the affiliation for Thomas T. Ibanez has been updated to “AMAP, University of Montpellier, CIRAD, CNRS, INRAE, IRD, Montpellier, France”, and the second affiliation for Sharif A. Mukul has been updated to “Department of Environment and Development Studies, United International University, Dhaka, Bangladesh”. The corrections have been made to the HTML and PDF versions of the article.

