# Supplementary material for: Native diversity buffers against severity of non-native tree invasions
Source: Nature. 2023 Aug 23;621(7980):773–81. doi: 10.1038/s41586-023-06440-7 (PMC10533391; doi:10.1038/s41586-023-06440-7)
Supplement: Supplementary file 1 — This file contains Supplementary Tables 1–9. [file 41586_2023_6440_MOESM1_ESM.docx]

**Native diversity buffers against severity of non-native tree invasions**

**Supplementary Information Guide**

***SI Table 1 | Summary statistics of study data***

| **A. Full matched dataset** | | | | | |
| --- | --- | --- | --- | --- | --- |
|  | Number of invaded plots | Number of non-invaded plots | Number of total plots | Proportion plots invaded | Mean proportion plot invaded |
| **i. All plots** | | | | | |
| Global | 22,994 | 448,894 | 471,888 | 0.049 | 0.24 |
| **ii. Bioclimatic Zones** | | | | | |
| Tropical | 565 | 3,153 | 3,718 | 0.152 | 0.202 |
| Temperate | 21,635 | 391,856 | 413,491 | 0.052 | 0.239 |
| **iii. Biome** | | | | | |
| Boreal | 22 | 42624 | 42,646 | 0.001 | 0.252 |
| Flooded Grasslands | 14 | 104 | 118 | 0.119 | 0.274 |
| Mangroves | 5 | 18 | 23 | 0.217 | 0.309 |
| Mediterranean woodlands | 709 | 8475 | 9,184 | 0.077 | 0.287 |
| Montane Grasslands | 0 | 70 | 70 | 0.000 | NA |
| Temperate BroadLeaf | 15921 | 302309 | 318,230 | 0.050 | 0.261 |
| Temperate Coniferous | 1784 | 39628 | 41,412 | 0.043 | 0.214 |
| Temperate Grasslands | 3930 | 49919 | 53,849 | 0.073 | 0.164 |
| Tropical Coniferous | 0 | 538 | 538 | 0.000 | NA |
| Tropical Deciduous Broadleaf | 61 | 183 | 244 | 0.250 | 0.361 |
| Tropical Grasslands | 138 | 544 | 682 | 0.202 | 0.205 |
| Tropical Moist Broadleaf | 366 | 1888 | 2,254 | 0.162 | 0.175 |
| Tundra | 26 | 828 | 854 | 0.030 | 0.257 |
| Xeric shrublands | 18 | 1766 | 1,784 | 0.010 | 0.279 |
| **B. Down-sampled dataset** | | | | | |
| **i. All plots** | | | | | |
| Global | 3,516 | 14,222 | 17,738 | 0.198 | 0.180 |
| **ii. Bioclimatic Zones** | | | | | |
| Tropical | 492 | 2,706 | 3,198 | 0.154 | 0.157 |
| Temperate | 2,719 | 3,971 | 6,690 | 0.406 | 0.175 |
| **iii. Biome** | | | | | |
| Boreal | 14 | 4322 | 4,336 | 0.003 | 0.205 |
| Flooded Grasslands | 4 | 104 | 108 | 0.037 | 0.133 |
| Mangroves | 3 | 18 | 21 | 0.143 | 0.292 |
| Mediterranean woodlands | 251 | 466 | 717 | 0.350 | 0.218 |
| Montane Grasslands | 0 | 52 | 52 | 0.000 | NA |
| Temperate BroadLeaf | 1113 | 1871 | 2,984 | 0.373 | 0.183 |
| Temperate Coniferous | 393 | 561 | 954 | 0.412 | 0.183 |
| Temperate Grasslands | 1213 | 1539 | 2,752 | 0.441 | 0.164 |
| Tropical Coniferous | 0 | 169 | 169 | 0.000 | NA |
| Tropical Deciduous Broadleaf | 38 | 111 | 149 | 0.255 | 0.265 |
| Tropical Grasslands | 112 | 544 | 656 | 0.171 | 0.167 |
| Tropical Moist Broadleaf | 342 | 1882 | 2,224 | 0.154 | 0.142 |
| Tundra | 18 | 817 | 835 | 0.022 | 0.213 |
| Xeric shrublands | 15 | 1766 | 1,781 | 0.008 | 0.227 |

***SI Table 2 | List of non-native tree species***

| Species | Occurrence Frequency |
| --- | --- |
| **A. Full matched dataset** | |
| *Robinia pseudoacacia* | 3976 |
| *Pinus sylvestris* | 2603 |
| *Maclura pomifera* | 2493 |
| *Picea abies* | 2468 |
| *Ailanthus altissima* | 1597 |
| *Pseudotsuga menziesii* | 1451 |
| *Castanea sativa* | 1132 |
| *Abies concolor* | 1067 |
| *Triadica sebifera* | 917 |
| *Morus alba* | 910 |
| *Quercus rubra* | 910 |
| *Eucalyptus globulus* | 753 |
| *Picea sitchensis* | 744 |
| *Pinus radiata* | 679 |
| *Prunus serotina* | 590 |
| *Melia azedarach* | 520 |
| *Larix decidua* | 429 |
| *Ulmus pumila* | 410 |
| *Paulownia tomentosa* | 373 |
| *Abies alba* | 315 |
| *Pinus mugo* | 256 |
| *Abies grandis* | 241 |
| *Pinus contorta* | 233 |
| *Albizia julibrissin* | 227 |
| *Pyrus communis* | 227 |
| *Picea glauca* | 215 |
| *Prunus avium* | 202 |
| *Abies nordmanniana* | 185 |
| *Pinus strobus* | 180 |
| *Acer platanoides* | 156 |
| *Leucaena leucocephala* | 134 |
| *Carya illinoinensis* | 132 |
| *Spathodea campanulata* | 122 |
| *Pinus nigra* | 114 |
| *Pinus elliottii* | 108 |
| *Pinus resinosa* | 92 |
| *Magnolia grandiflora* | 90 |
| *Acer negundo* | 79 |
| *Catalpa speciosa* | 73 |
| *Juglans regia* | 71 |
| *Syzygium jambos* | 65 |
| *Hovenia dulcis* | 63 |
| *Pinus taeda* | 62 |
| *Larix kaempferi* | 61 |
| *Picea pungens* | 56 |
| *Prunus persica* | 53 |
| *Psidium cattleianum* | 53 |
| *Aesculus hippocastanum* | 52 |
| *Chamaecyparis lawsoniana* | 49 |
| *Eucalyptus camaldulensis* | 49 |
| *Cedrus atlantica* | 47 |
| *Elaeagnus angustifolia* | 47 |
| *Pinus halepensis* | 44 |
| *Mangifera indica* | 41 |
| *Salix alba* | 34 |
| *Prunus domestica* | 30 |
| *Prosopis pallida* | 29 |
| *Vernicia fordii* | 29 |
| *Catalpa bignonioides* | 28 |
| *Melaleuca quinquenervia* | 28 |
| *Melicoccus bijugatus* | 27 |
| *Cinnamomum camphora* | 25 |
| *Albizia procera* | 24 |
| *Prunus virginiana* | 24 |
| *Acacia melanoxylon* | 23 |
| *Persea americana* | 23 |
| *Acacia dealbata* | 22 |
| *Alnus glutinosa* | 22 |
| *Cocos nucifera* | 21 |
| *Coffea arabica* | 21 |
| *Erythrina poeppigiana* | 20 |
| *Acer pseudoplatanus* | 19 |
| *Prunus cerasifera* | 19 |
| *Albizia lebbeck* | 18 |
| *Eucalyptus gomphocephala* | 18 |
| *Ficus carica* | 18 |
| *Psidium guajava* | 17 |
| *Schinus terebinthifolia* | 16 |
| *Alnus incana* | 15 |
| *Populus alba* | 14 |
| *Vitex parviflora* | 14 |
| *Podocarpus latifolius* | 13 |
| *Syzygium cumini* | 13 |
| *Artocarpus altilis* | 12 |
| *Eucalyptus robusta* | 12 |
| *Ziziphus jujuba* | 12 |
| *Pithecellobium dulce* | 11 |
| *Quercus bicolor* | 11 |
| *Senna siamea* | 11 |
| *Terminalia catappa* | 11 |
| *Coffea liberica* | 10 |
| *Gleditsia triacanthos* | 10 |
| *Carya laciniosa* | 9 |
| *Cecropia obtusifolia* | 9 |
| *Delonix regia* | 9 |
| *Pinus pinaster* | 9 |
| *Populus trichocarpa* | 9 |
| *Aleurites moluccanus* | 8 |
| *Magnolia obovata* | 8 |
| *Tamarindus indica* | 8 |
| *Carica papaya* | 7 |
| *Ceratonia siliqua* | 7 |
| *Gliricidia sepium* | 7 |
| *Castanea mollissima* | 6 |
| *Grevillea robusta* | 6 |
| *Juglans nigra* | 6 |
| *Pinus banksiana* | 6 |
| *Pinus virginiana* | 6 |
| *Platanus occidentalis* | 6 |
| *Swietenia macrophylla* | 6 |
| *Tsuga heterophylla* | 6 |
| *Adenanthera pavonina* | 5 |
| *Eriobotrya japonica* | 5 |
| *Morinda citrifolia* | 5 |
| *Piper aduncum* | 5 |
| *Ricinus communis* | 5 |
| *Toona ciliata* | 5 |
| *Alnus cordata* | 4 |
| *Andira inermis* | 4 |
| *Ardisia elliptica* | 4 |
| *Areca catechu* | 4 |
| *Bischofia javanica* | 4 |
| *Hibiscus elatus* | 4 |
| *Massularia acuminata* | 4 |
| *Morus nigra* | 4 |
| *Pinus ponderosa* | 4 |
| *Populus deltoides* | 4 |
| *Populus nigra* | 4 |
| *Prunus cerasus* | 4 |
| *Schefflera actinophylla* | 4 |
| *Sorbus aucuparia* | 4 |
| *Triplaris cumingiana* | 4 |
| *Acacia confusa* | 3 |
| *Acer rubrum* | 3 |
| *Artocarpus heterophyllus* | 3 |
| *Casuarina equisetifolia* | 3 |
| *Citharexylum caudatum* | 3 |
| *Cordia sebestena* | 3 |
| *Dillenia indica* | 3 |
| *Fagus sylvatica* | 3 |
| *Hakea sericea* | 3 |
| *Melochia umbellata* | 3 |
| *Pinus caribaea* | 3 |
| *Quercus alba* | 3 |
| *Quercus macrocarpa* | 3 |
| *Quercus palustris* | 3 |
| *Rhamnus cathartica* | 3 |
| *Sterculia apetala* | 3 |
| *Theobroma cacao* | 3 |
| *Annona muricata* | 2 |
| *Annona reticulata* | 2 |
| *Archontophoenix alexandrae* | 2 |
| *Ardisia solanacea* | 2 |
| *Bauhinia monandra* | 2 |
| *Betula populifolia* | 2 |
| *Caesalpinia pulcherrima* | 2 |
| *Carya texana* | 2 |
| *Cercis canadensis* | 2 |
| *Cladrastis kentukea* | 2 |
| *Clitoria fairchildiana* | 2 |
| *Cochlospermum vitifolium* | 2 |
| *Cordyline fruticosa* | 2 |
| *Crataegus azarolus* | 2 |
| *Crataegus monogyna* | 2 |
| *Erythrina abyssinica* | 2 |
| *Eucalyptus deglupta* | 2 |
| *Eugenia uniflora* | 2 |
| *Ficus microcarpa* | 2 |
| *Fraxinus pennsylvanica* | 2 |
| *Fraxinus uhdei* | 2 |
| *Ginkgo biloba* | 2 |
| *Haematoxylum campechianum* | 2 |
| *Inga nobilis* | 2 |
| *Lawsonia inermis* | 2 |
| *Morella faya* | 2 |
| *Quercus texana* | 2 |
| *Solanum mauritianum* | 2 |
| *Sorbus aria* | 2 |
| *Spondias dulcis* | 2 |
| *Spondias purpurea* | 2 |
| *Syzygium malaccense* | 2 |
| *Tecoma stans* | 2 |
| *Thuja occidentalis* | 2 |
| *Triphasia trifolia* | 2 |
| *Abies balsamea* | 1 |
| *Abies pinsapo* | 1 |
| *Acacia mearnsii* | 1 |
| *Aesculus glabra* | 1 |
| *Annona squamosa* | 1 |
| *Averrhoa bilimbi* | 1 |
| *Bauhinia multinervia* | 1 |
| *Berberis glaucocarpa* | 1 |
| *Betula papyrifera* | 1 |
| *Bixa orellana* | 1 |
| *Cananga odorata* | 1 |
| *Canarium harveyi* | 1 |
| *Carya ovata* | 1 |
| *Cassia fistula* | 1 |
| *Castilla elastica* | 1 |
| *Casuarina cristata* | 1 |
| *Casuarina glauca* | 1 |
| *Cecropia peltata* | 1 |
| *Ceiba pentandra* | 1 |
| *Chione venosa* | 1 |
| *Chrysophyllum oliviforme* | 1 |
| *Cinnamomum verum* | 1 |
| *Clusia rosea* | 1 |
| *Cordia dichotoma* | 1 |
| *Dillenia suffruticosa* | 1 |
| *Dracaena fragrans* | 1 |
| *Eucalyptus saligna* | 1 |
| *Fraxinus excelsior* | 1 |
| *Funtumia elastica* | 1 |
| *Gmelina arborea* | 1 |
| *Hakea salicifolia* | 1 |
| *Heterospathe elata* | 1 |
| *Inocarpus fagifer* | 1 |
| *Juglans hindsii* | 1 |
| *Leptospermum scoparium* | 1 |
| *Ligustrum lucidum* | 1 |
| *Macaranga mappa* | 1 |
| *Magnolia champaca* | 1 |
| *Magnolia fraseri* | 1 |
| *Magnolia virginiana* | 1 |
| *Manilkara zapota* | 1 |
| *Moringa oleifera* | 1 |
| *Nephelium lappaceum* | 1 |
| *Peltophorum pterocarpum* | 1 |
| *Pimenta dioica* | 1 |
| *Pinus monophylla* | 1 |
| *Plumeria rubra* | 1 |
| *Polyscias fruticosa* | 1 |
| *Pouteria sapota* | 1 |
| *Prunus debilis* | 1 |
| *Prunus laurocerasus* | 1 |
| *Prunus mahaleb* | 1 |
| *Pterocarpus macrocarpus* | 1 |
| *Quercus laurifolia* | 1 |
| *Ravenala madagascariensis* | 1 |
| *Rhus typhina* | 1 |
| *Salix cinerea* | 1 |
| *Sambucus nigra* | 1 |
| *Sambucus racemosa* | 1 |
| *Senna spectabilis* | 1 |
| *Solanum torvum* | 1 |
| *Syzygium samarangense* | 1 |
| *Taxodium distichum* | 1 |
| *Trema orientalis* | 1 |
| *Ulmus minor* | 1 |
| **B. Down-sampled dataset** | |
| *Maclura pomifera* | 717 |
| *Robinia pseudoacacia* | 557 |
| *Triadica sebifera* | 408 |
| *Picea abies* | 325 |
| *Abies concolor* | 302 |
| *Pinus sylvestris* | 299 |
| *Castanea sativa* | 258 |
| *Ailanthus altissima* | 237 |
| *Morus alba* | 210 |
| *Melia azedarach* | 192 |
| *Pseudotsuga menziesii* | 161 |
| *Leucaena leucocephala* | 134 |
| *Spathodea campanulata* | 122 |
| *Ulmus pumila* | 111 |
| *Quercus rubra* | 109 |
| *Pinus radiata* | 87 |
| *Picea sitchensis* | 82 |
| *Eucalyptus globulus* | 77 |
| *Syzygium jambos* | 65 |
| *Larix decidua* | 64 |
| *Hovenia dulcis* | 63 |
| *Paulownia tomentosa* | 58 |
| *Albizia julibrissin* | 57 |
| *Psidium cattleianum* | 53 |
| *Prunus serotina* | 45 |
| *Mangifera indica* | 41 |
| *Pinus elliottii* | 39 |
| *Prunus avium* | 37 |
| *Pinus contorta* | 34 |
| *Abies alba* | 31 |
| *Pinus halepensis* | 29 |
| *Prosopis pallida* | 29 |
| *Pyrus communis* | 29 |
| *Acer platanoides* | 28 |
| *Juglans regia* | 28 |
| *Catalpa speciosa* | 27 |
| *Melicoccus bijugatus* | 27 |
| *Carya illinoinensis* | 25 |
| *Albizia procera* | 24 |
| *Persea americana* | 23 |
| *Eucalyptus camaldulensis* | 22 |
| *Melaleuca quinquenervia* | 22 |
| *Cocos nucifera* | 21 |
| *Coffea arabica* | 21 |
| *Erythrina poeppigiana* | 20 |
| *Picea glauca* | 20 |
| *Pinus strobus* | 20 |
| *Abies grandis* | 19 |
| *Cedrus atlantica* | 19 |
| *Pinus mugo* | 19 |
| *Acer pseudoplatanus* | 18 |
| *Albizia lebbeck* | 18 |
| *Magnolia grandiflora* | 17 |
| *Psidium guajava* | 17 |
| *Schinus terebinthifolia* | 16 |
| *Pinus taeda* | 15 |
| *Ficus carica* | 14 |
| *Pinus resinosa* | 14 |
| *Vitex parviflora* | 14 |
| *Podocarpus latifolius* | 13 |
| *Abies nordmanniana* | 12 |
| *Artocarpus altilis* | 12 |
| *Eucalyptus robusta* | 12 |
| *Syzygium cumini* | 12 |
| *Ziziphus jujuba* | 12 |
| *Pithecellobium dulce* | 11 |
| *Senna siamea* | 11 |
| *Terminalia catappa* | 11 |
| *Coffea liberica* | 10 |
| *Acer negundo* | 9 |
| *Cecropia obtusifolia* | 9 |
| *Cinnamomum camphora* | 9 |
| *Delonix regia* | 9 |
| *Aesculus hippocastanum* | 8 |
| *Aleurites moluccanus* | 8 |
| *Pinus nigra* | 8 |
| *Salix alba* | 8 |
| *Tamarindus indica* | 8 |
| *Acacia dealbata* | 7 |
| *Carica papaya* | 7 |
| *Gliricidia sepium* | 7 |
| *Ceratonia siliqua* | 6 |
| *Elaeagnus angustifolia* | 6 |
| *Grevillea robusta* | 6 |
| *Prunus domestica* | 6 |
| *Prunus virginiana* | 6 |
| *Swietenia macrophylla* | 6 |
| *Vernicia fordii* | 6 |
| *Adenanthera pavonina* | 5 |
| *Catalpa bignonioides* | 5 |
| *Eriobotrya japonica* | 5 |
| *Morinda citrifolia* | 5 |
| *Picea pungens* | 5 |
| *Piper aduncum* | 5 |
| *Ricinus communis* | 5 |
| *Toona ciliata* | 5 |
| *Alnus glutinosa* | 4 |
| *Andira inermis* | 4 |
| *Ardisia elliptica* | 4 |
| *Areca catechu* | 4 |
| *Bischofia javanica* | 4 |
| *Hibiscus elatus* | 4 |
| *Larix kaempferi* | 4 |
| *Massularia acuminata* | 4 |
| *Morus nigra* | 4 |
| *Pinus pinaster* | 4 |
| *Prunus persica* | 4 |
| *Quercus bicolor* | 4 |
| *Schefflera actinophylla* | 4 |
| *Triplaris cumingiana* | 4 |
| *Acacia confusa* | 3 |
| *Artocarpus heterophyllus* | 3 |
| *Casuarina equisetifolia* | 3 |
| *Chamaecyparis lawsoniana* | 3 |
| *Citharexylum caudatum* | 3 |
| *Cordia sebestena* | 3 |
| *Dillenia indica* | 3 |
| *Melochia umbellata* | 3 |
| *Pinus caribaea* | 3 |
| *Populus alba* | 3 |
| *Sterculia apetala* | 3 |
| *Theobroma cacao* | 3 |
| *Acacia melanoxylon* | 2 |
| *Annona muricata* | 2 |
| *Annona reticulata* | 2 |
| *Archontophoenix alexandrae* | 2 |
| *Ardisia solanacea* | 2 |
| *Bauhinia monandra* | 2 |
| *Caesalpinia pulcherrima* | 2 |
| *Clitoria fairchildiana* | 2 |
| *Cochlospermum vitifolium* | 2 |
| *Cordyline fruticosa* | 2 |
| *Erythrina abyssinica* | 2 |
| *Eucalyptus deglupta* | 2 |
| *Eugenia uniflora* | 2 |
| *Ficus microcarpa* | 2 |
| *Fraxinus uhdei* | 2 |
| *Haematoxylum campechianum* | 2 |
| *Inga nobilis* | 2 |
| *Lawsonia inermis* | 2 |
| *Morella faya* | 2 |
| *Populus trichocarpa* | 2 |
| *Quercus alba* | 2 |
| *Spondias dulcis* | 2 |
| *Spondias purpurea* | 2 |
| *Syzygium malaccense* | 2 |
| *Tecoma stans* | 2 |
| *Thuja occidentalis* | 2 |
| *Triphasia trifolia* | 2 |
| *Alnus incana* | 1 |
| *Annona squamosa* | 1 |
| *Averrhoa bilimbi* | 1 |
| *Bauhinia multinervia* | 1 |
| *Bixa orellana* | 1 |
| *Cananga odorata* | 1 |
| *Canarium harveyi* | 1 |
| *Carya laciniosa* | 1 |
| *Cassia fistula* | 1 |
| *Castilla elastica* | 1 |
| *Casuarina cristata* | 1 |
| *Casuarina glauca* | 1 |
| *Cecropia peltata* | 1 |
| *Ceiba pentandra* | 1 |
| *Chione venosa* | 1 |
| *Chrysophyllum oliviforme* | 1 |
| *Cinnamomum verum* | 1 |
| *Cladrastis kentukea* | 1 |
| *Clusia rosea* | 1 |
| *Cordia dichotoma* | 1 |
| *Crataegus azarolus* | 1 |
| *Crataegus monogyna* | 1 |
| *Dillenia suffruticosa* | 1 |
| *Dracaena fragrans* | 1 |
| *Eucalyptus gomphocephala* | 1 |
| *Eucalyptus saligna* | 1 |
| *Fagus sylvatica* | 1 |
| *Fraxinus excelsior* | 1 |
| *Funtumia elastica* | 1 |
| *Gleditsia triacanthos* | 1 |
| *Gmelina arborea* | 1 |
| *Heterospathe elata* | 1 |
| *Inocarpus fagifer* | 1 |
| *Juglans nigra* | 1 |
| *Leptospermum scoparium* | 1 |
| *Ligustrum lucidum* | 1 |
| *Macaranga mappa* | 1 |
| *Magnolia champaca* | 1 |
| *Manilkara zapota* | 1 |
| *Moringa oleifera* | 1 |
| *Nephelium lappaceum* | 1 |
| *Peltophorum pterocarpum* | 1 |
| *Pimenta dioica* | 1 |
| *Pinus banksiana* | 1 |
| *Pinus ponderosa* | 1 |
| *Plumeria rubra* | 1 |
| *Polyscias fruticosa* | 1 |
| *Pouteria sapota* | 1 |
| *Prunus cerasifera* | 1 |
| *Prunus debilis* | 1 |
| *Prunus laurocerasus* | 1 |
| *Pterocarpus macrocarpus* | 1 |
| *Quercus texana* | 1 |
| *Ravenala madagascariensis* | 1 |
| *Senna spectabilis* | 1 |
| *Solanum torvum* | 1 |
| *Syzygium samarangense* | 1 |
| *Taxodium distichum* | 1 |
| *Trema orientalis* | 1 |
| *Ulmus minor* | 1 |

***SI Table 3 | Model outputs for GLMs predicting invasion with phylogenetic diversity***

| **A. Global** | | | | |
| --- | --- | --- | --- | --- |
| Variable | Estimate | Std. Error | z value | p value |
| *Non-native presence (N = 17,640)* | | | | |
| (Intercept) | -1.7576044 | 0.0491151 | -35.785 | <2.00E-16 |
| Native Phylogenetic Richness (Faith’s PD) | -0.3443539 | 0.0433033 | -7.952 | 1.83E-15 |
| Native Phylogenetic Redundancy (MNTD) | -0.1256124 | 0.0302243 | -4.156 | 3.24E-05 |
| Distance to Ports | -0.0017959 | 0.0001567 | -11.461 | <2.00E-16 |
| Population Density | 0.0029977 | 0.0009903 | 3.027 | 0.00247 |
| Annual Mean Temperature | 1.0891269 | 0.0580432 | 18.764 | <2.00E-16 |
| Annual Mean Precipitation | -0.1718914 | 0.0611429 | -2.811 | 0.00493 |
| Absolute Depth to Bedrock | -0.1076238 | 0.0378441 | -2.844 | 0.00446 |
| Coarse Fragments | -0.5778853 | 0.0375773 | -15.379 | <2.00E-16 |
| Sand Content | 0.0836075 | 0.0497604 | 1.68 | 0.09292 |
| Silt Content | 0.857936 | 0.0499203 | 17.186 | <2.00E-16 |
| Soil pH H2O | 0.1996064 | 0.0476288 | 4.191 | 2.78E-05 |
| Spatial Autocovariate | 2.8643708 | 0.0574185 | 49.886 | <2.00E-16 |
| *Degree of Invasion* | | | | |
| *Non-native richness (N = 3,498)* | | | | |
| (Intercept) | -1.72E+00 | 3.51E-02 | -48.948 | <2e-16 |
| Native Phylogenetic Richness (Faith’s PD) | -9.49E-01 | 4.44E-02 | -2.14E+01 | <2e-16 |
| Native Phylogenetic Redundancy (MNTD) | -3.01E-01 | 2.16E-02 | -1.39E+01 | <2e-16 |
| Distance to Ports | -1.67E-04 | 9.88E-05 | -1.69E+00 | 0.0906 |
| Population Density | 5.21E-05 | 3.67E-04 | 1.42E-01 | 0.887 |
| Annual Mean Temperature | 4.21E-02 | 4.76E-02 | 8.85E-01 | 0.3764 |
| Annual Mean Precipitation | -4.93E-03 | 4.32E-02 | -1.14E-01 | 0.9092 |
| Absolute Depth to Bedrock | 9.75E-03 | 3.16E-02 | 3.08E-01 | 0.7579 |
| Coarse Fragments | 4.20E-02 | 2.66E-02 | 1.58E+00 | 0.114 |
| Sand Content | 4.17E-02 | 3.62E-02 | 1.15E+00 | 0.2495 |
| Silt Content | -5.22E-03 | 3.69E-02 | -1.42E-01 | 0.8874 |
| Soil pH H2O | 3.38E-02 | 3.54E-02 | 9.56E-01 | 0.3391 |
| Spatial Autocovariate | 1.06E+00 | 1.00E-01 | 1.05E+01 | <2e-16 |
| *Non-native abundance (N = 3,498)* | | | | |
| (Intercept) | -1.72E+00 | 3.51E-02 | -48.948 | <2e-16 |
| Native Phylogenetic Richness (Faith’s PD) | -9.49E-01 | 4.44E-02 | -2.14E+01 | <2e-16 |
| Native Phylogenetic Redundancy (MNTD) | -3.01E-01 | 2.16E-02 | -1.39E+01 | <2e-16 |
| Distance to Ports | -1.67E-04 | 9.88E-05 | -1.69E+00 | 0.0906 |
| Population Density | 5.21E-05 | 3.67E-04 | 1.42E-01 | 0.887 |
| Annual Mean Temperature | 4.21E-02 | 4.76E-02 | 8.85E-01 | 0.3764 |
| Annual Mean Precipitation | -4.93E-03 | 4.32E-02 | -1.14E-01 | 0.9092 |
| Absolute Depth to Bedrock | 9.75E-03 | 3.16E-02 | 3.08E-01 | 0.7579 |
| Coarse Fragments | 4.20E-02 | 2.66E-02 | 1.58E+00 | 0.114 |
| Sand Content | 4.17E-02 | 3.62E-02 | 1.15E+00 | 0.2495 |
| Silt Content | -5.22E-03 | 3.69E-02 | -1.42E-01 | 0.8874 |
| Soil pH H2O | 3.38E-02 | 3.54E-02 | 9.56E-01 | 0.3391 |
| Spatial Autocovariate | 1.06E+00 | 1.00E-01 | 1.05E+01 | <2e-16 |
| **B. Temperate** | | | | |
| *Non-native presence (N = 6,661)* | | | | |
| (Intercept) | -0.4221839 | 0.0537126 | -7.86 | 3.84E-15 |
| Native Phylogenetic Richness (Faith’s PD) | -0.0915917 | 0.0352011 | -2.602 | 0.009269 |
| Native Phylogenetic Redundancy (MNTD) | -0.1597517 | 0.0336315 | -4.75 | 2.03E-06 |
| Distance to Ports | -0.0005491 | 0.000175 | -3.137 | 0.001706 |
| Population Density | 0.0061128 | 0.0025148 | 2.431 | 0.015066 |
| Annual Mean Temperature | 0.5327138 | 0.0465366 | 11.447 | <2.00E-16 |
| Annual Mean Precipitation | -0.1592873 | 0.0461744 | -3.45 | 0.000561 |
| Absolute Depth to Bedrock | 0.0817585 | 0.0320318 | 2.552 | 0.010698 |
| Clay Content | 0.1873079 | 0.0401627 | 4.664 | 3.11E-06 |
| Soil pH H2O | 0.443441 | 0.0504278 | 8.794 | <2.00E-16 |
| Spatial Autocovariate | 2.6447978 | 0.0781525 | 33.842 | <2.00E-16 |
| *Degree of Invasion* | | | | |
| *Non-native richness (N = 2,710)* | | | | |
| (Intercept) | -1.62E+00 | 3.25E-02 | -49.959 | <2.00E-16 |
| Native Phylogenetic Richness (Faith’s PD) | -3.42E-01 | 2.39E-02 | -14.336 | <2.00E-16 |
| Native Phylogenetic Redundancy (MNTD) | -2.85E-01 | 2.27E-02 | -12.575 | <2.00E-16 |
| Distance to Ports | -1.37E-04 | 1.02E-04 | -1.339 | 0.180586 |
| Population Density | 5.71E-05 | 4.12E-04 | 0.139 | 0.889693 |
| Annual Mean Temperature | -5.69E-02 | 3.56E-02 | -1.597 | 0.110228 |
| Annual Mean Precipitation | -3.20E-02 | 3.04E-02 | -1.052 | 0.292824 |
| Absolute Depth to Bedrock | 3.50E-03 | 2.18E-02 | 0.161 | 0.872439 |
| Clay Content | -3.38E-02 | 2.63E-02 | -1.284 | 0.199198 |
| Soil pH H2O | -2.36E-02 | 3.32E-02 | -0.709 | 0.478312 |
| Spatial Autocovariate | 7.44E-01 | 2.04E-01 | 3.645 | 0.000267 |
| *Non-native abundance (N = 2,710)* | | | | |
| (Intercept) | -1.75E+00 | 3.48E-02 | -50.346 | <2.00E-16 |
| Native Phylogenetic Richness (Faith’s PD) | -9.86E-01 | 4.75E-02 | -20.745 | <2.00E-16 |
| Native Phylogenetic Redundancy (MNTD) | -3.17E-01 | 2.17E-02 | -14.641 | <2.00E-16 |
| Distance to Ports | -2.77E-04 | 8.44E-05 | -3.278 | 0.00105 |
| Population Density | 1.03E-04 | 3.59E-04 | 0.288 | 0.77349 |
| Annual Mean Temperature | 3.97E-02 | 4.06E-02 | 0.979 | 0.32779 |
| Annual Mean Precipitation | -9.20E-03 | 4.24E-02 | -0.217 | 0.82803 |
| Absolute Depth to Bedrock | 4.84E-03 | 3.04E-02 | 0.159 | 0.87335 |
| Clay Content | -3.93E-02 | 2.32E-02 | -1.695 | 0.09014 |
| Soil pH H2O | 2.81E-02 | 3.45E-02 | 0.815 | 0.41496 |
| Spatial Autocovariate | 3.84E-01 | 6.16E-02 | 6.229 | 4.71E-10 |
| **C. Tropical** | | | | |
| *Non-native presence (N = 3,177)* | | | | |
| (Intercept) | -2.3986675 | 0.2445764 | -9.807 | <2.00E-16 |
| Native Phylogenetic Richness (Faith’s PD) | -0.7016497 | 0.128499 | -5.46 | 4.75E-08 |
| Native Phylogenetic Redundancy (MNTD) | -0.2946713 | 0.0775698 | -3.799 | 0.000145 |
| Distance to Ports | -0.0025323 | 0.0006105 | -4.148 | 3.36E-05 |
| Population Density | 0.001946 | 0.0014905 | 1.306 | 0.191692 |
| Annual Mean Temperature | 0.6127102 | 0.1025669 | 5.974 | 2.32E-09 |
| Annual Mean Precipitation | 0.313928 | 0.1293696 | 2.427 | 0.015241 |
| Absolute Depth to Bedrock | -1.7412014 | 0.1487555 | -11.705 | <2.00E-16 |
| SOC Content | 0.0065312 | 0.0030185 | 2.164 | 0.030483 |
| Soil pH H2O | 0.0963999 | 0.1028277 | 0.937 | 0.348507 |
| Spatial Autocovariate | 2.6110674 | 0.169399 | 15.414 | <2.00E-16 |
| *Degree of Invasion* | | | | |
| *Non-native richness (N = 492)* | | | | |
| (Intercept) | -2.796235 | 0.21676 | -12.9 | <2.00E-16 |
| Native Phylogenetic Richness (Faith’s PD) | -1.6264947 | 0.1585931 | -10.256 | <2.00E-16 |
| Native Phylogenetic Redundancy (MNTD) | -0.2201646 | 0.0569835 | -3.864 | 0.000112 |
| Distance to Ports | -0.0019696 | 0.0006738 | -2.923 | 0.003467 |
| Population Density | 0.0006816 | 0.00136 | 0.501 | 0.616257 |
| Annual Mean Temperature | 0.3205307 | 0.095516 | 3.356 | 0.000791 |
| Annual Mean Precipitation | 0.2746788 | 0.1136294 | 2.417 | 0.015635 |
| Absolute Depth to Bedrock | 0.1924893 | 0.1527018 | 1.261 | 0.207469 |
| SOC Content | 0.0026363 | 0.0022395 | 1.177 | 0.239124 |
| Soil pH H2O | 0.1339022 | 0.0781437 | 1.714 | 0.086613 |
| Spatial Autocovariate | 1.0978654 | 0.1687236 | 6.507 | 7.67E-11 |
| *Non-native abundance (N = 492)* | | | | |
| (Intercept) | -2.9878535 | 0.2125684 | -14.056 | <2.00E-16 |
| Native Phylogenetic Richness (Faith’s PD) | -1.5414992 | 0.1638622 | -9.407 | <2.00E-16 |
| Native Phylogenetic Redundancy (MNTD) | -0.2569922 | 0.0571732 | -4.495 | 6.96E-06 |
| Distance to Ports | -0.001646 | 0.0006906 | -2.383 | 0.0172 |
| Population Density | 0.000946 | 0.0013638 | 0.694 | 0.4879 |
| Annual Mean Temperature | 0.4026926 | 0.094336 | 4.269 | 1.97E-05 |
| Annual Mean Precipitation | 0.126684 | 0.1113319 | 1.138 | 0.2552 |
| Absolute Depth to Bedrock | 0.2712338 | 0.14713 | 1.843 | 0.0653 |
| SOC Content | 0.0050755 | 0.0022236 | 2.283 | 0.0225 |
| Soil pH H2O | 0.078674 | 0.0779719 | 1.009 | 0.313 |
| Spatial Autocovariate | 0.1217208 | 0.1729327 | 0.704 | 0.4815 |

***SI Table 4 | Model outputs for GLMs predicting invasion with functional diversity***

| **A. Global** | | | | |
| --- | --- | --- | --- | --- |
| Variable | Estimate | Std. Error | z value | p value |
| *Non-native presence (N = 17,271)* | | | | |
| (Intercept) | -1.8021488 | 0.0498298 | -36.166 | <2.00E-16 |
| Native Functional Richness (Faith’s PD) | -0.3551706 | 0.0494252 | -7.186 | 6.67E-13 |
| Native Functional Redundancy (MNTD) | 0.0007742 | 0.031112 | 0.025 | 0.98015 |
| Distance to Ports | -0.0017149 | 0.0001572 | -10.908 | <2.00E-16 |
| Population Density | 0.0029558 | 0.0010758 | 2.747 | 0.00601 |
| Annual Mean Temperature | 1.0797043 | 0.0586398 | 18.412 | <2.00E-16 |
| Annual Mean Precipitation | -0.1382215 | 0.0612319 | -2.257 | 0.02399 |
| Absolute Depth to Bedrock | -0.1037398 | 0.0383719 | -2.704 | 0.00686 |
| Coarse Fragments | -0.5682383 | 0.0384648 | -14.773 | <2.00E-16 |
| Sand Content | 0.0551095 | 0.0496904 | 1.109 | 0.26741 |
| Silt Content | 0.8758918 | 0.0502428 | 17.433 | <2.00E-16 |
| Soil pH H2O | 0.2255035 | 0.0482828 | 4.67 | 3.01E-06 |
| Spatial Autocovariate | 2.8530446 | 0.0581081 | 49.099 | <2.00E-16 |
| *Degree of Invasion* | | | | |
| *Non-native richness (N = 3,368)* | | | | |
| (Intercept) | -1.80E+00 | 3.60E-02 | -5.01E+01 | <2e-16 |
| Native Functional Richness (Faith’s PD) | -1.29E+00 | 6.06E-02 | -2.13E+01 | <2e-16 |
| Native Functional Redundancy (MNTD) | -1.75E-01 | 2.06E-02 | -8.51E+00 | <2e-16 |
| Distance to Ports | -4.76E-05 | 9.99E-05 | -4.76E-01 | 0.6341 |
| Population Density | -4.22E-05 | 3.65E-04 | -1.16E-01 | 0.908 |
| Annual Mean Temperature | 1.16E-01 | 4.87E-02 | 2.38E+00 | 0.0174 |
| Annual Mean Precipitation | 1.80E-02 | 4.46E-02 | 4.03E-01 | 0.6872 |
| Absolute Depth to Bedrock | 1.86E-02 | 3.24E-02 | 5.74E-01 | 0.5658 |
| Coarse Fragments | 5.57E-02 | 2.78E-02 | 2.00E+00 | 0.0453 |
| Sand Content | 3.68E-02 | 3.65E-02 | 1.01E+00 | 0.3139 |
| Silt Content | 2.86E-02 | 3.77E-02 | 7.58E-01 | 0.4485 |
| Soil pH H2O | 6.32E-02 | 3.69E-02 | 1.71E+00 | 0.0869 |
| Spatial Autocovariate | 1.04E+00 | 1.05E-01 | 9.859 | <2e-16 |
| *Non-native abundance (N = 3,368)* | | | | |
| (Intercept) | -1.80E+00 | 3.57E-02 | -5.05E+01 | <2.00E-16 |
| Native Functional Richness (Faith’s PD) | -1.30E+00 | 6.49E-02 | -2.01E+01 | <2.00E-16 |
| Native Functional Redundancy (MNTD) | -1.69E-01 | 2.06E-02 | -8.23E+00 | <2.00E-16 |
| Distance to Ports | -1.80E-05 | 9.95E-05 | -1.81E-01 | 0.8563 |
| Population Density | -4.37E-05 | 3.54E-04 | -1.23E-01 | 0.9018 |
| Annual Mean Temperature | 1.18E-01 | 4.76E-02 | 2.47E+00 | 0.0136 |
| Annual Mean Precipitation | -2.81E-03 | 4.50E-02 | -6.20E-02 | 0.9503 |
| Absolute Depth to Bedrock | 2.65E-02 | 3.24E-02 | 8.17E-01 | 0.4139 |
| Coarse Fragments | 6.83E-02 | 2.74E-02 | 2.49E+00 | 0.0127 |
| Sand Content | 3.04E-02 | 3.62E-02 | 8.38E-01 | 0.4018 |
| Silt Content | 2.03E-02 | 3.69E-02 | 5.51E-01 | 0.5819 |
| Soil pH H2O | 4.15E-02 | 3.71E-02 | 1.12E+00 | 0.2632 |
| Spatial Autocovariate | 3.21E-01 | 6.93E-02 | 4.631 | 3.64E-06 |
| **B. Temperate** | | | | |
| *Non-native presence (N = 6,510)* | | | | |
| (Intercept) | -0.477638 | 0.0548247 | -8.712 | <2.00E-16 |
| Native Functional Richness (Faith’s PD) | -0.0560927 | 0.0327431 | -1.713 | 0.08669 |
| Native Functional Redundancy (MNTD) | -0.0112446 | 0.0333148 | -0.338 | 0.73572 |
| Distance to Ports | -0.0004704 | 0.0001776 | -2.649 | 0.00807 |
| Population Density | 0.0065468 | 0.002627 | 2.492 | 0.0127 |
| Annual Mean Temperature | 0.5501702 | 0.0468626 | 11.74 | <2.00E-16 |
| Annual Mean Precipitation | -0.0965439 | 0.0468658 | -2.06 | 0.0394 |
| Absolute Depth to Bedrock | 0.0891335 | 0.0323203 | 2.758 | 0.00582 |
| Clay Content | 0.2084254 | 0.0399802 | 5.213 | 1.86E-07 |
| Soil pH H2O | 0.4991194 | 0.0509522 | 9.796 | <2.00E-16 |
| Spatial Autocovariate | 2.6412709 | 0.0806263 | 32.759 | <2.00E-16 |
| *Degree of Invasion* | | | | |
| *Non-native richness (N = 2,603)* | | | | |
| (Intercept) | -1.66E+00 | 3.40E-02 | -48.826 | <2e-16 |
| Native Functional Richness (Faith’s PD) | -3.44E-01 | 2.27E-02 | -15.187 | <2e-16 |
| Native Functional Redundancy (MNTD) | -1.68E-01 | 2.02E-02 | -8.323 | <2e-16 |
| Distance to Ports | 6.13E-05 | 1.05E-04 | 0.585 | 0.558 |
| Population Density | -5.26E-05 | 4.07E-04 | -0.129 | 0.897 |
| Annual Mean Temperature | -3.91E-02 | 3.53E-02 | -1.108 | 0.268 |
| Annual Mean Precipitation | -6.23E-03 | 3.11E-02 | -0.2 | 0.842 |
| Absolute Depth to Bedrock | 3.27E-04 | 2.20E-02 | 0.015 | 0.988 |
| Clay Content | -1.91E-02 | 2.65E-02 | -0.721 | 0.471 |
| Soil pH H2O | -7.91E-03 | 3.42E-02 | -0.232 | 0.817 |
| Spatial Autocovariate | 3.03E-01 | 2.99E-01 | 1.013 | 0.311 |
| *Non-native abundance (N = 2,603)* | | | | |
| (Intercept) | -1.66E+00 | 3.40E-02 | -48.813 | <2e-16 |
| Native Functional Richness (Faith’s PD) | -3.45E-01 | 2.27E-02 | -15.236 | <2e-16 |
| Native Functional Redundancy (MNTD) | -1.68E-01 | 2.02E-02 | -8.315 | <2e-16 |
| Distance to Ports | 6.14E-05 | 1.05E-04 | 0.586 | 0.558 |
| Population Density | -5.28E-05 | 4.06E-04 | -0.13 | 0.897 |
| Annual Mean Temperature | -3.96E-02 | 3.53E-02 | -1.121 | 0.262 |
| Annual Mean Precipitation | -5.97E-03 | 3.12E-02 | -0.191 | 0.848 |
| Absolute Depth to Bedrock | -2.26E-04 | 2.21E-02 | -0.01 | 0.992 |
| Clay Content | -1.96E-02 | 2.65E-02 | -0.739 | 0.46 |
| Soil pH H2O | -9.33E-03 | 3.42E-02 | -0.272 | 0.785 |
| Spatial Autocovariate | 2.55E-02 | 9.37E-02 | 0.272 | 0.786 |
| **C. Tropical** | | | | |
| *Non-native presence (N = 3,130)* | | | | |
| (Intercept) | -2.3132441 | 0.2466968 | -9.377 | <2.00E-16 |
| Native Functional Richness (Faith’s PD) | -0.954306 | 0.1519927 | -6.279 | 3.42E-10 |
| Native Functional Redundancy (MNTD) | -0.002736 | 0.0957587 | -0.029 | 0.9772 |
| Distance to Ports | -0.0028939 | 0.0006231 | -4.644 | 3.42E-06 |
| Population Density | 0.0011985 | 0.0014183 | 0.845 | 0.3981 |
| Annual Mean Temperature | 0.6354482 | 0.1052812 | 6.036 | 1.58E-09 |
| Annual Mean Precipitation | 0.2483641 | 0.1292999 | 1.921 | 0.0548 |
| Absolute Depth to Bedrock | -1.7117254 | 0.1517035 | -11.283 | <2.00E-16 |
| SOC Content | 0.0053529 | 0.0030151 | 1.775 | 0.0758 |
| Soil pH H2O | 0.0810023 | 0.105725 | 0.766 | 0.4436 |
| Spatial Autocovariate | 2.7032424 | 0.1705003 | 15.855 | <2.00E-16 |
| *Degree of Invasion* | | | | |
| *Non-native richness (N = 478)* | | | | |
| (Intercept) | -2.8995393 | 0.2185242 | -13.269 | <2.00E-16 |
| Native Functional Richness (Faith’s PD) | -1.7574488 | 0.1862758 | -9.435 | <2.00E-16 |
| Native Functional Redundancy (MNTD) | -0.2466255 | 0.0790206 | -3.121 | 0.0018 |
| Distance to Ports | -0.0019207 | 0.0006927 | -2.773 | 0.00556 |
| Population Density | 0.0009891 | 0.0014018 | 0.706 | 0.48047 |
| Annual Mean Temperature | 0.4706571 | 0.0950619 | 4.951 | 7.38E-07 |
| Annual Mean Precipitation | 0.2678022 | 0.1213365 | 2.207 | 0.02731 |
| Absolute Depth to Bedrock | 0.2393189 | 0.1631367 | 1.467 | 0.14238 |
| SOC Content | 0.0028814 | 0.0023118 | 1.246 | 0.21262 |
| Soil pH H2O | 0.1668222 | 0.083597 | 1.996 | 0.04598 |
| Spatial Autocovariate | 1.0438146 | 0.1758148 | 5.937 | 2.90E-09 |
| *Non-native abundance (N = 478)* | | | | |
| (Intercept) | -2.9807786 | 0.2146129 | -13.889 | <2.00E-16 |
| Native Functional Richness (Faith’s PD) | -1.6781341 | 0.1906678 | -8.801 | <2.00E-16 |
| Native Functional Redundancy (MNTD) | -0.2668343 | 0.0797089 | -3.348 | 0.000815 |
| Distance to Ports | -0.001596 | 0.0006941 | -2.299 | 0.021484 |
| Population Density | 0.0016909 | 0.0014063 | 1.202 | 0.229233 |
| Annual Mean Temperature | 0.5028494 | 0.0944937 | 5.322 | 1.03E-07 |
| Annual Mean Precipitation | 0.1178366 | 0.1191714 | 0.989 | 0.322761 |
| Absolute Depth to Bedrock | 0.3357535 | 0.1563261 | 2.148 | 0.031732 |
| SOC Content | 0.004713 | 0.0023188 | 2.032 | 0.042105 |
| Soil pH H2O | 0.10052 | 0.083192 | 1.208 | 0.226936 |
| Spatial Autocovariate | 0.0348364 | 0.1711035 | 0.204 | 0.838667 |

***SI Table 5 | Model outputs for GLMs predicting invasion with species richness***

| **A. Global** | | | | |
| --- | --- | --- | --- | --- |
| Variable | Estimate | Std. Error | z value | p value |
| *Non-native presence (N = 17,640)* | | | | |
| (Intercept) | -1.5829239 | 0.0500128 | -31.65 | <2.00E-16 |
| log(native species richness) | -0.0200893 | 0.0026262 | -7.649 | 2.02E-14 |
| Distance to Ports | -0.0018132 | 0.0001567 | -11.574 | <2.00E-16 |
| Population Density | 0.002891 | 0.0009911 | 2.917 | 0.00354 |
| Annual Mean Temperature | 1.0725653 | 0.0577952 | 18.558 | <2.00E-16 |
| Annual Mean Precipitation | -0.1483597 | 0.0609942 | -2.432 | 0.015 |
| Absolute Depth to Bedrock | -0.0951221 | 0.0374849 | -2.538 | 0.01116 |
| Coarse Fragments | -0.5779105 | 0.0374575 | -15.428 | <2.00E-16 |
| Sand Content | 0.10036 | 0.0495037 | 2.027 | 0.04262 |
| Silt Content | 0.8681449 | 0.0496726 | 17.477 | <2.00E-16 |
| Soil pH H2O | 0.2324656 | 0.0472132 | 4.924 | 8.49E-07 |
| Spatial Autocovariate | 2.8326457 | 0.0564636 | 50.168 | <2.00E-16 |
| *Degree of Invasion* | | | | |
| *Non-native richness (N = 3,498)* | | | | |
| (Intercept) | -9.49E-01 | 4.21E-02 | -22.546 | <2e-16 |
| log(native species richness) | -1.04E-01 | 4.76E-03 | -21.794 | <2e-16 |
| Distance to Ports | -7.23E-05 | 9.89E-05 | -0.731 | 0.4646 |
| Population Density | -8.23E-05 | 3.70E-04 | -0.223 | 0.8238 |
| Annual Mean Temperature | 5.87E-02 | 4.76E-02 | 1.233 | 0.2176 |
| Annual Mean Precipitation | 6.47E-03 | 4.33E-02 | 0.149 | 0.8813 |
| Absolute Depth to Bedrock | 2.81E-02 | 3.16E-02 | 0.889 | 0.3742 |
| Coarse Fragments | 4.93E-02 | 2.66E-02 | 1.857 | 0.0633 |
| Sand Content | 8.09E-03 | 3.63E-02 | 0.223 | 0.8237 |
| Silt Content | -4.27E-02 | 3.70E-02 | -1.156 | 0.2478 |
| Soil pH H2O | 5.90E-02 | 3.51E-02 | 1.681 | 0.0927 |
| Spatial Autocovariate | 1.09E+00 | 8.74E-02 | 12.519 | <2e-16 |
| *Non-native abundance (N = 3,498)* | | | | |
| (Intercept) | -9.68E-01 | 4.62E-02 | -20.96 | <2.00E-16 |
| log(native species richness) | -1.02E-01 | 5.46E-03 | -18.742 | <2.00E-16 |
| Distance to Ports | -4.99E-05 | 9.84E-05 | -0.507 | 0.612 |
| Population Density | -1.16E-04 | 3.58E-04 | -0.322 | 0.7473 |
| Annual Mean Temperature | 5.91E-02 | 4.62E-02 | 1.278 | 0.2013 |
| Annual Mean Precipitation | -1.52E-02 | 4.39E-02 | -0.347 | 0.7288 |
| Absolute Depth to Bedrock | 3.26E-02 | 3.16E-02 | 1.031 | 0.3026 |
| Coarse Fragments | 5.76E-02 | 2.61E-02 | 2.206 | 0.0274 |
| Sand Content | 1.72E-02 | 3.61E-02 | 0.476 | 0.6338 |
| Silt Content | -3.36E-02 | 3.62E-02 | -0.929 | 0.3531 |
| Soil pH H2O | 4.73E-02 | 3.54E-02 | 1.337 | 0.1812 |
| Spatial Autocovariate | 4.40E-01 | 6.15E-02 | 7.15 | 8.70E-13 |
| **B. Temperate** | | | | |
| *Non-native presence (N = 6,661)* | | | | |
| (Intercept) | -0.3295764 | 0.0840252 | -3.922 | 8.77E-05 |
| log(native species richness) | -0.0143729 | 0.011695 | -1.229 | 0.219083 |
| Distance to Ports | -0.0005694 | 0.0001759 | -3.237 | 0.00121 |
| Population Density | 0.0061577 | 0.0025248 | 2.439 | 0.014735 |
| Annual Mean Temperature | 0.5371034 | 0.0456414 | 11.768 | <2.00E-16 |
| Annual Mean Precipitation | -0.1548299 | 0.0458951 | -3.374 | 0.000742 |
| Absolute Depth to Bedrock | 0.0886742 | 0.0319446 | 2.776 | 0.005505 |
| Clay Content | 0.1924706 | 0.0400005 | 4.812 | 1.50E-06 |
| Soil pH H2O | 0.4486488 | 0.0495566 | 9.053 | <2.00E-16 |
| Spatial Autocovariate | 2.6169141 | 0.0769027 | 34.029 | <2.00E-16 |
| *Degree of Invasion* | | | | |
| *Non-native richness (N = 2,710)* | | | | |
| (Intercept) | -6.97E-01 | 5.58E-02 | -12.482 | <2.00E-16 |
| log(native species richness) | -1.53E-01 | 8.09E-03 | -18.855 | <2.00E-16 |
| Distance to Ports | 6.10E-06 | 1.03E-04 | 0.059 | 0.9526 |
| Population Density | -9.27E-05 | 4.12E-04 | -0.225 | 0.82184 |
| Annual Mean Temperature | -2.84E-02 | 3.47E-02 | -0.817 | 0.4138 |
| Annual Mean Precipitation | -3.00E-02 | 3.03E-02 | -0.989 | 0.3225 |
| Absolute Depth to Bedrock | 2.14E-03 | 2.18E-02 | 0.098 | 0.92179 |
| Clay Content | -1.76E-02 | 2.63E-02 | -0.671 | 0.50191 |
| Soil pH H2O | -2.33E-02 | 3.28E-02 | -0.711 | 0.47714 |
| Spatial Autocovariate | 6.93E-01 | 2.37E-01 | 2.924 | 0.00346 |
| *Non-native abundance (N = 2,710)* | | | | |
| (Intercept) | -6.93E-01 | 5.61E-02 | -12.352 | <2e-16 |
| log(native species richness) | -1.54E-01 | 8.10E-03 | -19.015 | <2e-16 |
| Distance to Ports | 1.20E-05 | 1.03E-04 | 0.117 | 0.907 |
| Population Density | -1.02E-04 | 4.10E-04 | -0.248 | 0.804 |
| Annual Mean Temperature | -2.66E-02 | 3.46E-02 | -0.771 | 0.441 |
| Annual Mean Precipitation | -3.41E-02 | 3.03E-02 | -1.126 | 0.26 |
| Absolute Depth to Bedrock | 1.21E-03 | 2.19E-02 | 0.055 | 0.956 |
| Clay Content | -1.82E-02 | 2.63E-02 | -0.694 | 0.488 |
| Soil pH H2O | -2.96E-02 | 3.27E-02 | -0.905 | 0.365 |
| Spatial Autocovariate | 1.14E-01 | 8.25E-02 | 1.382 | 0.167 |
| **C. Tropical** | | | | |
| *Non-native presence (N = 3,177)* | | | | |
| (Intercept) | -1.7641839 | 0.241688 | -7.299 | 2.89E-13 |
| log(native species richness) | -0.0240154 | 0.0032864 | -7.308 | 2.72E-13 |
| Distance to Ports | -0.0026496 | 0.0006085 | -4.354 | 1.34E-05 |
| Population Density | 0.0017407 | 0.0014977 | 1.162 | 0.24513 |
| Annual Mean Temperature | 0.6865046 | 0.1037291 | 6.618 | 3.63E-11 |
| Annual Mean Precipitation | 0.32638 | 0.125932 | 2.592 | 0.00955 |
| Absolute Depth to Bedrock | -1.6896133 | 0.144577 | -11.687 | <2.00E-16 |
| SOC Content | 0.0074345 | 0.0029435 | 2.526 | 0.01155 |
| Soil pH H2O | 0.1284637 | 0.1007866 | 1.275 | 0.20245 |
| Spatial Autocovariate | 2.5985514 | 0.1666951 | 15.589 | <2.00E-16 |
| *Degree of Invasion* | | | | |
| *Non-native richness (N = 492)* | | | | |
| (Intercept) | -1.5539396 | 0.2171384 | -7.156 | 8.28E-13 |
| log(native species richness) | -0.0486852 | 0.0052595 | -9.257 | <2.00E-16 |
| Distance to Ports | -0.0023037 | 0.0007026 | -3.279 | 0.00104 |
| Population Density | 0.0006889 | 0.001335 | 0.516 | 0.60584 |
| Annual Mean Temperature | 0.3883841 | 0.0954342 | 4.07 | 4.71E-05 |
| Annual Mean Precipitation | 0.2757033 | 0.1102843 | 2.5 | 0.01242 |
| Absolute Depth to Bedrock | 0.3142942 | 0.1472066 | 2.135 | 0.03276 |
| SOC Content | 0.003794 | 0.0022398 | 1.694 | 0.09028 |
| Soil pH H2O | 0.2032197 | 0.07637 | 2.661 | 0.00779 |
| Spatial Autocovariate | 1.0367091 | 0.1436981 | 7.214 | 5.41E-13 |
|  |  |  |  |  |
| *Non-native abundance (N = 492)* | | | | |
| log(native species richness) | -0.046809 | 0.0056209 | -8.328 | <2.00E-16 |
| Distance to Ports | -0.0018434 | 0.0007034 | -2.621 | 0.00878 |
| Population Density | 0.0007453 | 0.0013355 | 0.558 | 0.57683 |
| Annual Mean Temperature | 0.4793327 | 0.0936065 | 5.121 | 3.04E-07 |
| Annual Mean Precipitation | 0.1599553 | 0.1083453 | 1.476 | 0.13985 |
| Absolute Depth to Bedrock | 0.4118596 | 0.1401183 | 2.939 | 0.00329 |
| SOC Content | 0.0068026 | 0.002195 | 3.099 | 0.00194 |
| Soil pH H2O | 0.1725286 | 0.0758293 | 2.275 | 0.02289 |
| Spatial Autocovariate | 0.2804694 | 0.1572274 | 1.784 | 0.07445 |

***SI Table 6 | Model outputs for GLMs predicting non-native invasion strategy***

| **A. Phylogenetic Invasion Strategy** | | | | |
| --- | --- | --- | --- | --- |
| **i. Global (N = 3,498; R-squared = 0.057, df = 3,486)** | | | | |
| Variable | Estimate | Std. Error | t value | p value |
| (Intercept) | -2.06E-02 | 1.30E-02 | -1.58E+00 | 1.14E-01 |
| Annual Mean Temperature (1) | 1.76E+00 | 2.35E-01 | 7.48E+00 | 9.69E-14 |
| Annual Mean Temperature (2) | -1.09E+01 | 1.62E+00 | -6.72E+00 | 2.13E-11 |
| Annual Mean Precipitation | 1.84E-01 | 1.63E-01 | 1.13E+00 | 2.59E-01 |
| Distance to Ports | -1.48E-04 | 3.70E-05 | -4.01E+00 | 6.21E-05 |
| Population Density | 1.76E-04 | 1.39E-04 | 1.27E+00 | 2.05E-01 |
| Native Phylogenetic Richness (Faith’s PD) | -1.12E-01 | 1.39E-02 | -8.06E+00 | 1.04E-15 |
| Absolute Depth to Bedrock | 2.78E-02 | 1.18E-02 | 2.36E+00 | 1.86E-02 |
| Coarse Fragments | -2.88E-03 | 9.90E-03 | -2.91E-01 | 7.71E-01 |
| Sand Content | 3.83E-04 | 6.96E-03 | 5.50E-02 | 9.56E-01 |
| Soil pH H2O | 9.54E-03 | 1.26E-02 | 7.60E-01 | 4.47E-01 |
| Spatial Autocovariate | 5.98E-01 | 6.22E-02 | 9.615 | <2.00E-16 |
| **ii. Temperate (N = 2,710; R-squared = 0.120, df = 2,699)** | | | | |
| (Intercept) | -9.87E-03 | 9.57E-03 | -1.03E+00 | 0.30218 |
| Annual Mean Temperature | 5.96E-01 | 1.32E-01 | 4.52E+00 | 6.38E-06 |
| Annual Mean Precipitation | -1.17E-01 | 8.56E-02 | -1.37E+00 | 0.171806 |
| Distance to Ports | -2.04E-04 | 3.27E-05 | -6.24E+00 | 5.06E-10 |
| Population Density | 1.42E-04 | 1.17E-04 | 1.21E+00 | 0.224948 |
| Native Phylogenetic Richness (Faith’s PD) | -8.48E-02 | 6.66E-03 | -1.27E+01 | <2.00E-16 |
| Absolute Depth to Bedrock | 1.04E-02 | 6.27E-03 | 1.65E+00 | 0.09908 |
| Clay Content | 1.67E-02 | 7.60E-03 | 2.20E+00 | 0.028042 |
| Soil pH H2O | 7.87E-03 | 9.57E-03 | 8.23E-01 | 0.41083 |
| Annual Mean Temperature* Distance to Ports | 2.28E-03 | 5.91E-04 | 3.859 | 0.000116 |
| Spatial Autocovariate | 6.91E-01 | 5.87E-02 | 11.776 | <2.00E-16 |
| **iii. Tropical (N = 492; R-squared = 0.0291, df = 482)** | | | | |
| (Intercept) | 1.05E-01 | 1.31E-01 | 8.02E-01 | 0.4228 |
| Annual Mean Temperature | -9.59E-01 | 5.22E-01 | -1.84E+00 | 0.0667 |
| Annual Mean Precipitation | -6.72E-01 | 6.74E-01 | -9.98E-01 | 0.319 |
| Distance to Ports | -5.59E-05 | 3.24E-04 | -1.72E-01 | 0.8632 |
| Population Density | 1.58E-03 | 1.03E-03 | 1.54E+00 | 0.1255 |
| Native Phylogenetic Richness (Faith’s PD) | -2.57E-02 | 6.77E-02 | -3.79E-01 | 0.705 |
| Absolute Depth to Bedrock | 1.39E-01 | 9.97E-02 | 1.40E+00 | 0.1638 |
| SOC Content | -4.18E-04 | 1.32E-03 | -3.18E-01 | 0.751 |
| Soil pH H2O | -1.88E-02 | 4.96E-02 | -3.78E-01 | 0.7056 |
| Spatial Autocovariate | -3.35E-01 | 3.63E-01 | -0.921 | 0.3573 |
| **B. Functional Introduction Strategy** | | | | |
| **i. Global (N = 3,368; R-squared = 0.149, df = 3,356)** | | | | |
| Variable | Estimate | Std. Error | t value | p value |
| (Intercept) | -4.58E-02 | 3.74E-03 | -1.23E+01 | <2.00E-16 |
| Annual Mean Temperature (1) | 5.43E-01 | 6.49E-02 | 8.37E+00 | <2.00E-16 |
| Annual Mean Temperature (2) | -1.76E+00 | 4.52E-01 | -3.88E+00 | 1.07E-04 |
| Annual Mean Precipitation | 5.43E-01 | 6.49E-02 | 8.37E+00 | <2.00E-16 |
| Distance to Ports | 2.07E-05 | 1.05E-05 | 1.97E+00 | 4.88E-02 |
| Population Density | -2.98E-05 | 3.93E-05 | -7.59E-01 | 0.447972 |
| Native Functional Richness (Faith’s) | -1.46E-02 | 4.50E-03 | -3.24E+00 | 0.00121 |
| Absolute Depth to Bedrock | 1.97E-03 | 3.37E-03 | 5.84E-01 | 0.559308 |
| Coarse Fragments | -1.46E-02 | 2.86E-03 | -5.11E+00 | 3.37E-07 |
| Sand Content | -7.14E-03 | 2.00E-03 | -3.57E+00 | 0.00036 |
| Soil pH H2O | 1.27E-02 | 3.64E-03 | 3.50E+00 | 0.000479 |
| Spatial Autocovariate | 7.14E-01 | 4.70E-02 | 1.52E+01 | <2.00E-16 |
| **ii. Temperate (N = 2,603; R-squared = 0.167, df = 2,592)** | | | | |
| (Intercept) | -3.43E-02 | 3.47E-03 | -9.88E+00 | <2.00E-16 |
| Annual Mean Temperature | 4.19E-01 | 3.56E-02 | 1.18E+01 | <2.00E-16 |
| Annual Mean Precipitation | -1.94E-01 | 3.67E-02 | -5.28E+00 | 1.41E-07 |
| Distance to Ports | 6.36E-05 | 1.12E-05 | 5.68E+00 | 1.48E-08 |
| Population Density | -1.38E-05 | 4.19E-05 | -3.29E-01 | 0.742121 |
| Native Functional Richness (Faith’s) | -1.21E-02 | 2.34E-03 | -5.17E+00 | 2.48E-07 |
| Absolute Depth to Bedrock | 2.28E-03 | 2.25E-03 | 1.01E+00 | 0.312321 |
| Clay Content | 7.71E-03 | 2.70E-03 | 2.85E+00 | 0.004369 |
| Soil pH H2O | 1.22E-02 | 3.54E-03 | 3.46E+00 | 0.000553 |
| Annual Mean Precipitation* Distance to Ports | 8.45E-04 | 1.15E-04 | 7.347 | 2.71E-13 |
| Spatial Autocovariate | 7.57E-01 | 5.25E-02 | 14.412 | <2.00E-16 |
| **iii. Tropical (N = 478; R-squared = 0.057, df = 467)** | | | | |
| (Intercept) | 2.01E-03 | 1.82E-02 | 1.10E-01 | 0.912182 |
| Annual Mean Temperature | -1.28E-01 | 6.92E-02 | -1.84E+00 | 0.06605 |
| Annual Mean Precipitation | -4.13E-03 | 9.46E-02 | -4.40E-02 | 0.965201 |
| Distance to Ports | -2.94E-06 | 4.46E-05 | -6.60E-02 | 0.947597 |
| Population Density | 2.17E-04 | 1.36E-04 | 1.59E+00 | 0.112532 |
| Native Functional Richness (Faith’s) | 4.36E-03 | 1.00E-02 | 4.35E-01 | 0.663683 |
| Absolute Depth to Bedrock | 1.41E-03 | 1.39E-02 | 1.02E-01 | 0.918982 |
| SOC Content | -9.33E-05 | 1.79E-04 | -5.20E-01 | 0.603023 |
| Soil pH H2O | 2.63E-02 | 6.74E-03 | 3.897 | 0.000112 |
| Annual Mean Temperature * Annual Mean Precipitation | 1.71E+00 | 9.55E-01 | 1.786 | 0.074682 |
| Spatial Autocovariate | 1.73E-01 | 1.86E-01 | 0.931 | 0.352116 |

***SI Table 7 | Model outputs for GLMs predicting invasion with phylogenetic and functional diversity using intact plots only***

| **A. Phylogenetic Diversity** | | | | |
| --- | --- | --- | --- | --- |
| Variable | Estimate | Std. Error | z value | p value |
| *Non-native presence (N = 1,536)* | | | | |
| (Intercept) | -4.504993 | 0.454905 | -9.903 | <2.00E-16 |
| Native Phylogenetic Richness (Faith’s PD) | -0.300867 | 2.62E-01 | -1.149 | 2.50E-01 |
| Native Phylogenetic Redundancy (MNTD) | 0.222257 | 2.44E-01 | 0.911 | 3.62E-01 |
| Distance to Ports | -0.001168 | 1.29E-03 | -0.905 | 0.365496 |
| Population Density | 1.52E-01 | 0.040483 | 3.766 | 1.66E-04 |
| Annual Mean Temperature | 1.457268 | 6.53E-01 | 2.231 | 0.025654 |
| Annual Mean Precipitation | -0.275896 | 4.02E-01 | -0.686 | 4.93E-01 |
| Absolute Depth to Bedrock | -1.165462 | 0.669158 | -1.742 | 8.16E-02 |
| Coarse Fragments | -6.34E-01 | 0.343585 | -1.845 | 6.50E-02 |
| Sand Content | 4.33E-01 | 0.243322 | 1.78 | 0.075131 |
| Silt Content | 9.15E-01 | 0.448708 | 2.04 | 0.041383 |
| Soil pH H2O | 0.236995 | 3.84E-01 | 0.617 | 5.37E-01 |
| Spatial Autocovariate | 2.339296 | 0.29901 | 7.823 | 5.14E-15 |
| **B. Functional Diversity** | | | | |
| *Non-native presence (N = 1,510)* | | | | |
| (Intercept) | -4.553576 | 0.469367 | -9.702 | <2.00E-16 |
| Native Functional Richness (Faith’s PD) | -0.160331 | 0.279824 | -0.573 | 0.566664 |
| Native Functional Redundancy (MNTD) | 0.187479 | 0.237228 | 0.79 | 0.429357 |
| Distance to Ports | -0.001133 | 0.001309 | -0.865 | 0.387003 |
| Population Density | 0.135428 | 0.03827 | 3.539 | 0.000402 |
| Annual Mean Temperature | 1.382754 | 0.664712 | 2.08 | 0.037504 |
| Annual Mean Precipitation | -0.241003 | 0.40655 | -0.593 | 0.553315 |
| Absolute Depth to Bedrock | -1.017393 | 0.676643 | -1.504 | 0.132687 |
| Coarse Fragments | -0.499507 | 0.357243 | -1.398 | 0.162044 |
| Sand Content | 0.239104 | 0.255588 | 0.936 | 0.349528 |
| Silt Content | 0.964288 | 0.446683 | 2.159 | 0.030868 |
| Soil pH H2O | 0.227825 | 0.398619 | 0.572 | 0.567636 |
| Spatial Autocovariate | 2.284371 | 0.285782 | 7.993 | 1.31E-15 |

***SI Table 8 | Model outputs for GLMs predicting invasion with phylogenetic and function diversity using data down-sampled without preferentially retaining invaded plots***

| **A. Phylogenetic Diversity** | | | | |
| --- | --- | --- | --- | --- |
| Variable | Estimate | Std. Error | z value | p value |
| *Non-native presence (N = 18,898)* | | | | |
| (Intercept) | -3.590342 | 0.0885943 | -40.526 | <2.00E-16 |
| Native Phylogenetic Richness (Faith’s PD) | -0.2232515 | 5.02E-02 | -4.446 | 8.73E-06 |
| Native Phylogenetic Redundancy (MNTD) | -0.0975927 | 5.36E-02 | -1.82 | 6.87E-02 |
| Distance to Ports | -0.0020585 | 2.50E-04 | -8.242 | <2.00E-16 |
| Population Density | 2.23E-03 | 0.0012645 | 1.764 | 7.78E-02 |
| Annual Mean Temperature | 0.8353995 | 8.62E-02 | 9.686 | <2.00E-16 |
| Annual Mean Precipitation | 0.3792668 | 7.33E-02 | 5.171 | 2.32E-07 |
| Absolute Depth to Bedrock | -0.2671307 | 0.0614687 | -4.346 | 1.39E-05 |
| Coarse Fragments | -5.06E-01 | 0.0644234 | -7.851 | 4.13E-15 |
| Sand Content | -6.58E-01 | 0.0636861 | -10.332 | <2.00E-16 |
| Silt Content | 1.34E-01 | 0.0603451 | 2.228 | 0.0258 |
| Soil pH H2O | 0.4317014 | 7.31E-02 | 5.904 | 3.55E-09 |
| Spatial Autocovariate | 2.9325991 | 0.126298 | 23.22 | <2.00E-16 |
| *Degree of Invasion* | | | | |
| *Non-native richness (N = 840)* | | | | |
| (Intercept) | -1.8436494 | 0.0944234 | -19.525 | <2.00E-16 |
| Native Phylogenetic Richness (Faith’s PD) | -0.8838088 | 0.0647795 | -13.643 | <2.00E-16 |
| Native Phylogenetic Redundancy (MNTD) | -0.3178436 | 0.050176 | -6.335 | 2.38E-10 |
| Distance to Ports | -0.0005455 | 0.0002425 | -2.249 | 0.0245 |
| Population Density | 0.0009991 | 0.0012932 | 0.773 | 0.4398 |
| Annual Mean Temperature | 0.1633768 | 0.0856736 | 1.907 | 0.0565 |
| Annual Mean Precipitation | 0.0666511 | 0.066066 | 1.009 | 0.313 |
| Absolute Depth to Bedrock | 0.0274407 | 0.0637495 | 0.43 | 0.6669 |
| Coarse Fragments | 0.0436683 | 0.0575184 | 0.759 | 0.4477 |
| Sand Content | 0.0951116 | 0.0590539 | 1.611 | 0.1073 |
| Silt Content | 0.1115699 | 0.0562916 | 1.982 | 0.0475 |
| Soil pH H2O | 0.1322331 | 0.0740038 | 1.787 | 0.074 |
| Spatial Autocovariate | 1.0406296 | 0.1168259 | 8.908 | <2.00E-16 |
| *Non-native abundance (N = 840)* | | | | |
| (Intercept) | -1.8551373 | 0.0954978 | -19.426 | <2.00E-16 |
| Native Phylogenetic Richness (Faith’s PD) | -0.8941932 | 0.070215 | -12.735 | <2.00E-16 |
| Native Phylogenetic Redundancy (MNTD) | -0.3377648 | 0.050573 | -6.679 | 2.41E-11 |
| Distance to Ports | -0.0004657 | 0.0002438 | -1.91 | 0.0561 |
| Population Density | 0.0018269 | 0.0012985 | 1.407 | 0.1594 |
| Annual Mean Temperature | 0.1843009 | 0.0830581 | 2.219 | 0.0265 |
| Annual Mean Precipitation | 0.0384857 | 0.0658501 | 0.584 | 0.5589 |
| Absolute Depth to Bedrock | 0.0528897 | 0.063184 | 0.837 | 0.4026 |
| Coarse Fragments | 0.0978343 | 0.0556174 | 1.759 | 0.0786 |
| Sand Content | 0.0896508 | 0.0579602 | 1.547 | 0.1219 |
| Silt Content | 0.1179076 | 0.0546514 | 2.157 | 0.031 |
| Soil pH H2O | 0.1122124 | 0.0734196 | 1.528 | 0.1264 |
| Spatial Autocovariate | 0.5478533 | 0.1188581 | 4.609 | 4.04E-06 |
| **B. Functional Diversity** | | | | |
| *Non-native presence (N = 18,611)* | | | | |
| (Intercept) | -3.6218134 | 9.03E-02 | -40.097 | <2.00E-16 |
| Native Functional Richness (Faith’s PD) | -0.2360682 | 5.54E-02 | -4.259 | 2.06E-05 |
| Native Functional Redundancy (MNTD) | -0.0583639 | 5.38E-02 | -1.086 | 2.78E-01 |
| Distance to Ports | -2.02E-03 | 0.0002494 | -8.099 | 5.52E-16 |
| Population Density | 0.0020011 | 1.22E-03 | 1.638 | 1.01E-01 |
| Annual Mean Temperature | 0.852519 | 8.76E-02 | 9.731 | <2.00E-16 |
| Annual Mean Precipitation | 0.3668423 | 0.0719499 | 5.099 | 3.42E-07 |
| Absolute Depth to Bedrock | -2.64E-01 | 0.0624073 | -4.225 | 2.38E-05 |
| Coarse Fragments | -5.02E-01 | 0.0652259 | -7.699 | 1.37E-14 |
| Sand Content | -6.91E-01 | 0.0642804 | -10.752 | <2.00E-16 |
| Silt Content | 0.1305473 | 6.05E-02 | 2.157 | 0.031 |
| Soil pH H2O | 0.4331969 | 0.0743966 | 5.823 | 5.79E-09 |
| Spatial Autocovariate | 2.8916345 | 0.1292885 | 22.366 | <2.00E-16 |
| *Degree of Invasion* | | | | |
| *Non-native richness (N = 823)* | | | | |
| (Intercept) | -1.9660445 | 0.0970893 | -20.25 | <2.00E-16 |
| Native Functional Richness (Faith’s PD) | -1.0604242 | 0.0773154 | -13.716 | <2.00E-16 |
| Native Functional Redundancy (MNTD) | -0.1904755 | 0.0490201 | -3.886 | 0.000102 |
| Distance to Ports | -0.0004413 | 0.0002454 | -1.798 | 0.07213 |
| Population Density | 0.0012615 | 0.0013247 | 0.952 | 0.34095 |
| Annual Mean Temperature | 0.26994 | 0.0879797 | 3.068 | 0.002153 |
| Annual Mean Precipitation | 0.0883535 | 0.0678503 | 1.302 | 0.192854 |
| Absolute Depth to Bedrock | 0.0449774 | 0.0676534 | 0.665 | 0.506165 |
| Coarse Fragments | 0.0482035 | 0.0588551 | 0.819 | 0.412775 |
| Sand Content | 0.1049898 | 0.0594834 | 1.765 | 0.077559 |
| Silt Content | 0.1613935 | 0.0564083 | 2.861 | 0.004221 |
| Soil pH H2O | 0.2071737 | 0.0752921 | 2.752 | 0.00593 |
| Spatial Autocovariate | 1.0431751 | 0.1180789 | 8.835 | <2.00E-16 |
| *Non-native abundance (N = 823)* | | | | |
| (Intercept) | -1.9437467 | 0.0983326 | -19.767 | <2.00E-16 |
| Native Functional Richness (Faith’s PD) | -1.0521119 | 0.0854423 | -12.314 | <2.00E-16 |
| Native Functional Redundancy (MNTD) | -0.1783425 | 0.0490075 | -3.639 | 0.000274 |
| Distance to Ports | -0.0003405 | 0.0002462 | -1.383 | 0.166653 |
| Population Density | 0.0024192 | 0.0013395 | 1.806 | 0.070914 |
| Annual Mean Temperature | 0.2692905 | 0.0853021 | 3.157 | 0.001595 |
| Annual Mean Precipitation | 0.0552885 | 0.0676529 | 0.817 | 0.413793 |
| Absolute Depth to Bedrock | 0.0738926 | 0.0663986 | 1.113 | 0.265767 |
| Coarse Fragments | 0.1035553 | 0.0566186 | 1.829 | 0.0674 |
| Sand Content | 0.0894623 | 0.058706 | 1.524 | 0.127532 |
| Silt Content | 0.1478344 | 0.055451 | 2.666 | 0.007675 |
| Soil pH H2O | 0.1614709 | 0.0744729 | 2.168 | 0.030145 |
| Spatial Autocovariate | 0.4840084 | 0.1150662 | 4.206 | 2.60E-05 |
| Spatial Autocovariate | 0.4840084 | 0.1150662 | 4.206 | 2.60E-05 |

***SI Table 9 | Model outputs for GLMs predicting invasion with species richness corrected for invasion impact on species richness***

| Variable | Estimate | Std. Error | z value | p value |
| --- | --- | --- | --- | --- |
| *Corrected, mean (N = 17,640)* | | | | |
| *Estimated % biotic resistance attribuable to invasion:6.71* | | | | |
| (Intercept) | -1.22154 | 0.03746 | -32.60600 | <2.00E-16 |
| log(native species richness) | -0.02168 | 0.00247 | -8.78800 | <2.00E-16 |
| Distance to Ports | -0.00186 | 0.00012 | -15.64700 | <2.00E-16 |
| Population Density | 0.00728 | 0.00123 | 5.92400 | 0.00000 |
| Annual Mean Temperature | 0.78261 | 0.04078 | 19.19100 | <2.00E-16 |
| Annual Mean Precipitation | -0.05989 | 0.04302 | -1.39200 | 0.16386 |
| Absolute Depth to Bedrock | -0.06976 | 0.02915 | -2.39300 | 0.01673 |
| Coarse Fragments | -0.39890 | 0.02912 | -13.70100 | <2.00E-16 |
| Sand Content | -0.10463 | 0.03735 | -2.80200 | 0.00508 |
| Silt Content | 0.79855 | 0.03845 | 20.77100 | <2.00E-16 |
| Soil pH H2O | 0.32763 | 0.03603 | 9.09300 | <2.00E-16 |
| *Corrected, maximum 95% (N = 17,640)* | | | | |
| *Estimated % biotic resistance attribuable to invasion: 10.4* | | | | |
| (Intercept) | -1.22154 | 0.03746 | -32.60600 | <2.00E-16 |
| log(native species richness) | -0.02083 | 0.00237 | -8.78800 | <2.00E-16 |
| Distance to Ports | -0.00186 | 0.00012 | -15.64700 | <2.00E-16 |
| Population Density | 0.00728 | 0.00123 | 5.92400 | 0.00000 |
| Annual Mean Temperature | 0.78261 | 0.04078 | 19.19100 | <2.00E-16 |
| Annual Mean Precipitation | -0.05989 | 0.04302 | -1.39200 | 0.16386 |
| Absolute Depth to Bedrock | -0.06976 | 0.02915 | -2.39300 | 0.01673 |
| Coarse Fragments | -0.39890 | 0.02912 | -13.70100 | <2.00E-16 |
| Sand Content | -0.10463 | 0.03735 | -2.80200 | 0.00508 |
| Silt Content | 0.79855 | 0.03845 | 20.77100 | <2.00E-16 |
| Soil pH H2O | 0.32763 | 0.03603 | 9.09300 | <2.00E-16 |
| *Corrected, minimum 95% (N = 17,640)* | | | | |
| *Estimated % biotic resistance attribuable to invasion: 2.7* | | | | |
| (Intercept) | -1.22154 | 0.03746 | -32.60600 | <2.00E-16 |
| log(native species richness) | -0.02261 | 0.00257 | -8.78800 | <2.00E-16 |
| Distance to Ports | -0.00186 | 0.00012 | -15.64700 | <2.00E-16 |
| Population Density | 0.00728 | 0.00123 | 5.92400 | 0.00000 |
| Annual Mean Temperature | 0.78261 | 0.04078 | 19.19100 | <2.00E-16 |
| Annual Mean Precipitation | -0.05989 | 0.04302 | -1.39200 | 0.16386 |
| Absolute Depth to Bedrock | -0.06976 | 0.02915 | -2.39300 | 0.01673 |
| Coarse Fragments | -0.39890 | 0.02912 | -13.70100 | <2.00E-16 |
| Sand Content | -0.10463 | 0.03735 | -2.80200 | 0.00508 |
| Silt Content | 0.79855 | 0.03845 | 20.77100 | <2.00E-16 |
| Soil pH H2O | 0.32763 | 0.03603 | 9.09300 | <2.00E-16 |
