# Supplementary material for: Native diversity buffers against severity of non-native tree invasions
Source: Nature. 2023 Aug 23;621(7980):773–81. doi: 10.1038/s41586-023-06440-7 (PMC10533391; doi:10.1038/s41586-023-06440-7)
Supplement: Supplementary file 2 — Reporting Summary [file 41586_2023_6440_MOESM2_ESM.pdf]

nature portfolio

Corresponding author(s): Camille S. Delavaux

Last updated by author(s): Apr 15, 2023

Reporting Summary

Nature Portfolio wishes to improve the reproducibility of the work that we publish. This form provides structure for consistency and transparency in reporting. For further information on Nature Portfolio policies, see our Editorial Policies and the Editorial Policy Checklist.

Statistics

For all statistical analyses, confirm that the following items are present in the figure legend, table legend, main text, or Methods section.

n/a	Confirmed	
☐	☒	The exact sample size (*n*) for each experimental group/condition, given as a discrete number and unit of measurement
☒	☐	A statement on whether measurements were taken from distinct samples or whether the same sample was measured repeatedly
☐	☒	The statistical test(s) used AND whether they are one- or two-sided<br>*Only common tests should be described solely by name; describe more complex techniques in the Methods section.*
☐	☒	A description of all covariates tested
☐	☒	A description of any assumptions or corrections, such as tests of normality and adjustment for multiple comparisons
☐	☒	A full description of the statistical parameters including central tendency (e.g. means) or other basic estimates (e.g. regression coefficient) AND variation (e.g. standard deviation) or associated estimates of uncertainty (e.g. confidence intervals)
☐	☒	For null hypothesis testing, the test statistic (e.g. *F*, *t*, *r*) with confidence intervals, effect sizes, degrees of freedom and *P* value noted<br>*Give P values as exact values whenever suitable.*
☒	☐	For Bayesian analysis, information on the choice of priors and Markov chain Monte Carlo settings
☐	☒	For hierarchical and complex designs, identification of the appropriate level for tests and full reporting of outcomes
☒	☐	Estimates of effect sizes (e.g. Cohen's *d*, Pearson's *r*), indicating how they were calculated

Our web collection on statistics for biologists contains articles on many of the points above.

Software and code

Policy information about availability of computer code

Data collection	No software was used for data collection.
Data analysis	All code used to complete analyses for the manuscript is available at the following link: https://github.com/c383d893/GlobalInvasion.git. Data analysis was conducted in R (v. 4.2.2) and Python (v. 3.9.7), Google Earth Engine (earthengine-api 0.1.306), and on the ETH Zurich Euler cluster. R packages used in this study include tidyverse (v. 1.3.2), feather (v 0.3.5), doParallel (v. 1.0.17), foreach (v. 1.5.2), ape (v. 5.6-2), pez (v. 1.2-4), abdiv (v. 0.2.0), sp (v. 1.6.0), ncf (v. 1.3-2), spdep (v. 1.2-7), scales (v. 1.2.1), rsq (v. 2.5), geosphere (v. 1.5-18), lme4 (v. 1.1-31), lmerTest (v. 3.1-3), betareg (v. 3.1-4), ggeffects (v. 1.1.4), ggplot2 (v. 3.4.0), gridExtra (v. 2.3), grid (v. 4.2.2), ClustOfVar (v. 1.1), HH (v. 3.1-49), MuMIn (v. 1.47.1), viridis (v. 0.6.2), RColorBrewer (v. 1.1-3), fastshap (v. 0.0.7), ranger (v. 0.13.1), ggbeeswarm (v. 0.7.1), colorspace (v. 2.0-3), caret (v. 6.0-93), fmsb (v. 0.7.5), cowplot (v. 1.1.1), and AUC (v. 0.3.2). Python packages used in this study include numpy (v. 1.20.3), pandas (v. 1.5.3), scipy (v. 1.8.0), sklearn (v. 1.1.1), plotnine (v. 0.10.1), matplotlib (v. 3.5.1).

For manuscripts utilizing custom algorithms or software that are central to the research but not yet described in published literature, software must be made available to editors and reviewers. We strongly encourage code deposition in a community repository (e.g. GitHub). See the Nature Portfolio guidelines for submitting code & software for further information.

Data

Policy information about availability of data

All manuscripts must include a data availability statement. This statement should provide the following information, where applicable:

- Accession codes, unique identifiers, or web links for publicly available datasets
- A description of any restrictions on data availability
- For clinical datasets or third party data, please ensure that the statement adheres to our policy

Data used in this study can be found in cited references for the Global Naturalized Alien Flora (GloNAF) database6 (non-native status), the KEW Plants of the World database5 (native ranges), and the Global Environmental Composite65,82 (environmental data layers). Plant trait data were extracted from Maynard et al.83 Data from Global Forest Biodiversity Initiative (GFBI) database 59 are not available due to data privacy and sharing restrictions, but can be obtained upon request via Science-I (https://science-i.org/) or GFBI (htpps://www.gfbiinitiative.org/) and an approval from data contributors.

Human research participants

Policy information about studies involving human research participants and Sex and Gender in Research.

Reporting on sex and gender	N/A
Population characteristics	N/A
Recruitment	N/A
Ethics oversight	N/A

Note that full information on the approval of the study protocol must also be provided in the manuscript.

Field-specific reporting

Please select the one below that is the best fit for your research. If you are not sure, read the appropriate sections before making your selection.

☐ Life sciences ☐ Behavioural & social sciences ☒ Ecological, evolutionary & environmental sciences

For a reference copy of the document with all sections, see nature.com/documents/nr-reporting-summary-flat.pdf

Ecological, evolutionary & environmental sciences study design

All studies must disclose on these points even when the disclosure is negative.

Study description	We combined random forest and generalized linear model (GLM) approaches to answer our focal questions. Specifically, we used random forest models to visualize patterns and determine variable importance, while GLMs were used to assess statistical significance and directionality of patterns. We first tested for environmental and anthropogenic drivers of non-native invasion, including non-native presence and invasion severity (non-native richness, non-native abundance). Our independent variables included either phylogenetic or functional metrics, climate and soil variables, and human impact variables. Next, we tested the impact of these variables on non-native invasion strategy (difference in MNTD due to non-natives). We focused on addressing specific hypotheses related to drivers of non-native invasion and invasion strategy. We acknowledge the importance of other variables, and therefore included them in our models, but do not interpret each variable.
Research sample	The Global Forest Biodiversity Initiative (GFBI) database, which contains tree-level abundance data for more than 1.2 million forest plots on all continents across the globe, containing more than 31 million unique georeferenced records of tree size and density dating from 1958. Each observation in the dataset consists of a unique tree ID, plot ID, plot coordinates, tree diameter at breast height (DBH), tree-per-hectare expansion factors, year of measurement, and binomial species names.
Sampling strategy	To account for unequal representation of plots across biomes (Figure 1), we used a reduced version of this database, down-sampled to a number of plots proportional to the land area covered by each of 14 biomes (Table S1), while conserving as many tropical plots as possible. This ensured that we were not overrepresenting historically oversampled biomes (down-sampling), particularly in temperate regions. In addition, we preferentially retained invaded plots, or up-sampled to invasion within our down-sampling proportional to biome cover, to ensure that no more than half of the plots within a biome were invaded. This oversampling of invaded plots allowed for adequate representation of invaded and non-invaded plots in our analyses on non-native presence, and allowed sufficient data for our analyses of invasion severity, as these analyses only used data from plots that had non-native species invasions.
Data collection	Data collection varies across all datasets. Please refer to references for each dataset for more details.
Timing and spatial scale	Data timing and spatial scale varies across all datasets. Please refer to references for each dataset for more details.

Data exclusions	We applied several filters to this data before analyses. First, where plots had multiple years of data, we kept only the most recent year of census data. We then subset the data to include only plots with at least three species as required for our phylogenetic metrics, excluding monoculture forest plantations from the study.
Reproducibility	This study uses experimental data so was not possible to reproduce.
Randomization	Please see "Sampling strategy" for a detailed explanation of random subsampling.
Blinding	Blinding was not possible during data analysis.

Did the study involve field work? ☐ Yes ☒ No

Reporting for specific materials, systems and methods

We require information from authors about some types of materials, experimental systems and methods used in many studies. Here, indicate whether each material, system or method listed is relevant to your study. If you are not sure if a list item applies to your research, read the appropriate section before selecting a response.

Materials & experimental systems

n/a	Involved in the study
☒	☐ Antibodies
☒	☐ Eukaryotic cell lines
☒	☐ Palaeontology and archaeology
☒	☐ Animals and other organisms
☒	☐ Clinical data
☒	☐ Dual use research of concern

Methods

n/a	Involved in the study
☒	☐ ChIP-seq
☒	☐ Flow cytometry
☒	☐ MRI-based neuroimaging
